# Iron(II) Complexes of 2,6-Di[4-(ethylcarboxy)pyrazol-1-yl]pyridine with Reversible Guest-Modulated Spin-Crossover Behavior

**DOI:** 10.1021/acs.cgd.2c01524

**Published:** 2023-03-02

**Authors:** Víctor García-López, Hanane El Mansour El Jastimi, Jana Juráková, Miguel Clemente-León, Eugenio Coronado

**Affiliations:** † Instituto de Ciencia Molecular (ICMol), Universidad de Valencia, Catedrático José Beltrán 2, 46980 Paterna, Spain; ‡ Central European Institute of Technology, Brno University of Technology, Purkyn̆ova 123, 61200 Brno, Czech Republic

## Abstract

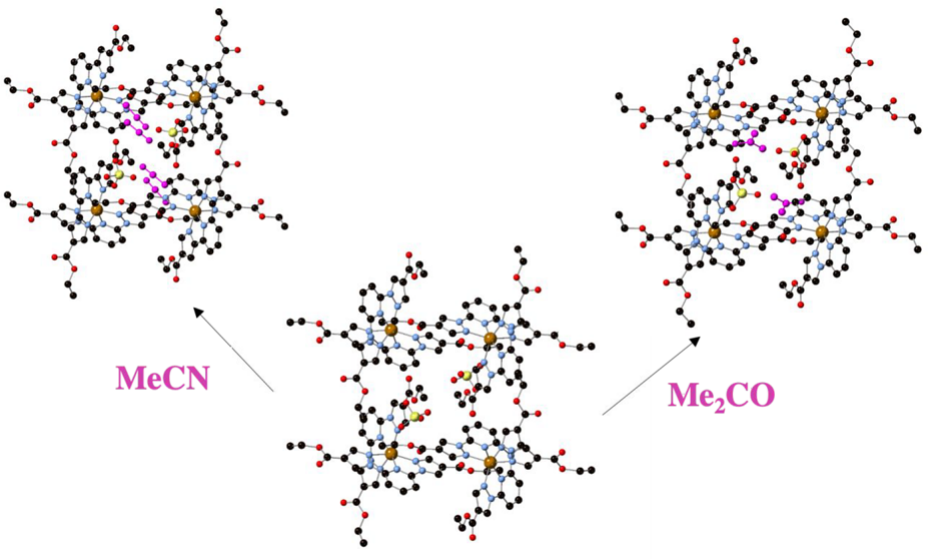

Three solvatomorphs of the iron(II) complex of 2,6-di[4-(ethylcarboxy)pyrazol-1-yl]pyridine (bpCOOEt_2_p) of formulas [Fe(bpCOOEt_2_p)_2_](ClO_4_)_2_·1.5MeNO_2_ (**1**), [Fe(bpCOOEt_2_p)_2_](ClO_4_)_2_·MeNO_2_ (**2**), and [Fe(bpCOOEt_2_p)_2_](ClO_4_)_2_·2MeNO_2_ (**3**) have been prepared and characterized. They show interesting spin-crossover (SCO) properties ranging from partial to complete thermal spin transitions and a light-induced excited spin-state trapping (LIESST) effect. In solvatomorph **2**, a robust structure is formed with channels that enable the entrance or removal of solvent molecules by vapor diffusion without losing the crystallinity. Thus, solvent-exchanged samples [Fe(bpCOOEt_2_p)_2_](ClO_4_)_2_·MeNO_2_ (**2·MeNO**_**2**_), [Fe(bpCOOEt_2_p)_2_](ClO_4_)_2_·MeCN (**2·MeCN**), [Fe(bpCOOEt_2_p)_2_](ClO_4_)_2_·0.5Me_2_CO **(2·Me**_**2**_**CO**), and [Fe(bpCOOEt_2_p)_2_](ClO_4_)_2_·MeCOOH (**2·MeCOOH**) were prepared by vapor diffusion of the solvents in a crystal of the compound previously heated to 400 K in a single-crystal to single-crystal (SCSC) fashion. Interestingly, this causes a change of spin state with a stabilization of the low-spin state in **2·Me**_**2**_**CO** and the high-spin state in **2·MeCN**. Therefore, the SCO properties of **2** can be tuned in a reversible way by exposure to different solvents.

## Abstract 2

Three solvatomorphs of the iron(II) complex of bpCOOEt_2_p have been prepared and characterized. In one of them, a robust structure is formed enabling the entrance or removal of solvent molecules by vapor diffusion without losing the crystallinity. This allows a reversible tuning of the spin-crossover properties.

## Introduction

Spin-crossover (SCO) complexes, which can be reversibly switched between the low-spin (LS) state and the high-spin (HS) state by external stimuli such as light, temperature, pressure, electric field or guest molecules, constitute one of the best examples of molecular bistability.^1,2^ Due to this, they are being proposed for an increasing number of applications such as sensors, memory devices or spintronic devices.^3−6^ In particular, they are appealing systems for sensing applications since some of their properties such as color or magnetism are highly sensitive to their environment and, hence, to the reversible absorption of guest molecules such as gas or solvent molecules. Many examples of guest-sensitive SCO compounds have been reported in polymeric porous coordination polymers or metal–organic frameworks (MOFs), as they contain extended networks of chemical bonds and pores that prevent the network from collapsing after removal of the guest molecules and provide a pathway for the loss and uptake of the solvent molecules.^7^ However, in the last few years several molecular-based SCO compounds have been reported to undergo absorption, desorption, or substitution of solvent molecules coupled to a change of their spin state, which can be accompanied by single-crystal to single-crystal (SCSC) transformations.^8−18^

**Chart 1:**
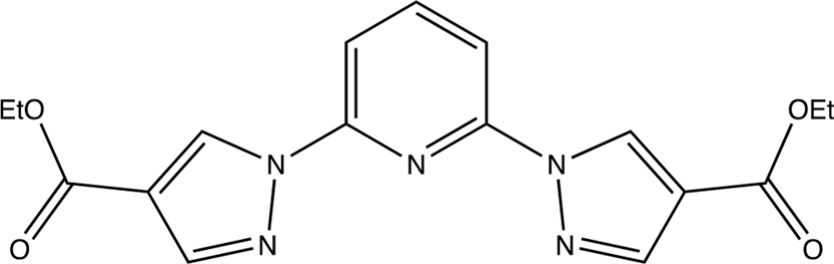
Molecular Structure of bpCOOEt_2_p

Fe(II) bis-chelated complexes based on 2,6-bis(pyrazol-1-yl)pyridine (bpp) represent one of the most studied families of SCO compounds.^19−22^ They usually display abrupt thermal and light-induced (known as light-induced excited spin-state trapping, LIESST) spin transitions at relatively high temperatures.^23−25^ In addition, they are also very versatile ligands since the addition of functional groups in different positions of the ligand is feasible without perturbing the SCO properties. Their magnetic properties are also very sensitive to the presence of solvent molecules, which in some cases are accompanied by SCSC transformations as in the [FeL_2_][BF_4_]_2_ compound with L = 4-(isopropylsulfanyl)-2,6-di(pyrazol-1-yl)pyridine.^26^ The preparation of Fe(II) complexes of bpp derivatives functionalized in the 4-pyrazolyl positions has been less explored. In one case, the incorporation of carboxylic acid at these positions afforded an Fe(II) SCO complex with an abrupt and hysteretic thermal spin transition close to room temperature associated with a crystallographic phase transition and a LIESST effect with an unexpectedly high *T*(LIESST) of 120 K.^27^ In this case, the combination of the rigidity provided by the tridentate bpp ligand together with the flexibility associated with the presence of counteranions and solvent molecules allowed a huge structural reorganization of the molecules accompanying the spin transition. In this work we have attempted the formation of flexible structures based on similar complexes replacing the carboxylic acid derivative by a carboxylic ethyl ester derivative in a 2,6-di[4-(ethylcarboxy)pyrazol-1-yl]pyridine (bpCOOEt_2_p, see Chart 1) ligand, which could be an additional source of flexibility and interesting SCO properties. Halcrow et al. reported the iron(II) complex of this ligand in [Fe(bpCOOEt_2_p)_2_](BF_4_)_2_·CF_3_CH_2_OH compound, which remained in the LS state at all temperatures.^28^ In this work, we have explored the preparation of nitromethane (MeNO_2_) solvates of this complex. This has given rise to three different solvatomorphs displaying gradual thermal spin transitions and a LIESST effect. In addition, in one of these MeNO_2_ solvates, the flexibility afforded by the carboxylic ethyl ester substituents gave rise to SCSC transformations accompanying the loss of the MeNO_2_ solvent molecules and the absorption by vapor diffusion of different solvent molecules (MeNO_2_, Me_2_CO, MeCN, and MeCOOH). This allowed reversible tuning of the spin state of the iron(II) complexes.

## Experimental Section

### Synthesis

BpCOOEt_2_p was prepared as reported in the literature.^28^ All other chemicals are commercially available and were used as received without further purification.

### [Fe(bpCOOEt_2_p)_2_](ClO_4_)_2_·1.5MeNO_2_ (**1**), [Fe(bpCOOEt_2_p)_2_](ClO_4_)_2_·MeNO_2_ (**2**), and [Fe(bpCOOEt_2_p)_2_](ClO_4_)_2_·2MeNO_2_ (**3**)

**CAUTION: perchlorate salts are explosive when heated or subjected to friction**.

Fe(ClO_4_)_2_·*x*H_2_O (36 mg, 0.05 mmol) in MeNO_2_ (3 mL) was added to a solution of bpCOOEtp (64.4 mg, 0.10 mmol) in MeNO_2_ (3 mL) inside the N_2_ atmosphere of a glovebox. The mixture was stirred for 15 min. A yellow solution was obtained. Yellow prismatic crystals of [Fe(bpCOOEt_2_p)_2_](ClO_4_)_2_·1.5MeNO_2_ (**1**) suitable for X-ray diffraction were obtained by slow liquid diffusion of diethyl ether into this solution in a thick tube (diameter 1 cm) after 1 week inside the glovebox. If the diffusion tubes were left undisturbed after this time, the crystals of **1** dissolved, and orange rectangular crystals of **2** suitable for X-ray diffraction appeared at the bottom part of the tube after a few weeks. Vapor-to-liquid diffusion of diethyl ether into the MeNO_2_ solution gives rise to **3**. The composition of crystals of all the complexes was checked by microanalysis and shows a Fe:Cl ratio close to 1:2. Elemental analysis of filtered samples of all the complexes is consistent with the presence of water molecules. This suggests loss of solvent molecules and absorption of water molecules after filtering the crystals. Anal. Calcd for Fe(N_5_C_17_H_17_O_4_)_2_(ClO_4_)_2_(H_2_O)_2_ (filtered sample of **1**): C, 40.78; H, 3.82; N, 13.99%. Found: C, 40.93; H, 3.81; N, 13.72%. Anal. Calcd for Fe(N_5_C_17_H_17_O_4_)_2_(ClO_4_)_2_(H_2_O)_3_·(CH_3_NO_2_)_0.5_ (filtered sample of **2**): C, 39.48; H, 3.98; N, 14.02%. Found: C, 39.31; H, 3.55; N, 13.89%. Anal. Calcd for Fe(N_5_C_17_H_17_O_4_)_2_(ClO_4_)_2_(H_2_O)_4_·(CH_3_NO_2_)_0.5_ (filtered sample of **3**): C, 38.80; H, 4.10; N, 13.77%. Found: C, 38.23; H, 3.69; N, 13.38%.

### Solvent-Exchanged Samples [Fe(bpCOOEt_2_p)_2_](ClO_4_)_2_·MeNO_2_ (**2·MeNO_2_**), [Fe(bpCOOEt_2_p)_2_](ClO_4_)_2_·MeCN (**2·MeCN**), [Fe(bpCOOEt_2_p)_2_](ClO_4_)_2_·0.5Me_2_CO (**2·Me_2_CO**), and [Fe(bpCOOEt_2_p)_2_](ClO_4_)_2_·MeCOOH (**2·MeCOOH**)

Single crystals of solvent exchanged samples were prepared by introducing a single crystal previously desolvated at 400 K in the diffractometer into a saturated atmosphere of MeNO_2_, MeCN, Me_2_CO, or MeCOOH for a few hours. Resolvated polycrystalline samples were prepared by depositing a desolvated polycrystalline sample in the bottom of a glass tube, which was kept in contact with a saturated atmosphere of the solvents for 24 h. After this time, the sample was quickly sealed and measured in the same tube used as a sample holder. The same sample was used for all the magnetic measurements of the exchanged samples.

### Structural Characterization

Single crystals of all complexes were mounted on a glass fiber using a viscous hydrocarbon oil to coat the crystal and then transferred directly to the cold nitrogen stream for data collection. X-ray data were collected at different temperatures on a Supernova diffractometer equipped with a graphite-monochromated Enhance (Mo) X-ray Source (λ = 0.71073 Å). The program CrysAlisPro, Oxford Diffraction Ltd., was used for unit cell determinations and data reduction. Empirical absorption correction was performed using spherical harmonics, implemented in the SCALE3 ABSPACK scaling algorithm. The structures were solved with the ShelXT structure solution program^29^ and refined with the SHELXL-2013 program,^30^ using Olex2.^31^ Non-hydrogen atoms were refined anisotropically, and hydrogen atoms were placed in calculated positions refined using idealized geometries (riding model) and assigned fixed isotropic displacement parameters. Crystallographic data are summarized in Table S1. CCDC 2230342–2230352 contain the supplementary crystallographic data for this paper. These data can be obtained free of charge from The Cambridge Crystallographic Data Centre via www.ccdc.cam.ac.uk/data_request/cif. For X-ray powder pattern, a 0.7 mm glass capillary was filled with a polycrystalline sample of the complexes and mounted and aligned on an Empyrean PANalytical powder diffractometer, using CuKα radiation (λ = 1.54177 Å). A total of 3 scans were collected at room temperature in the 2θ range 5–40°.

### Physical Characterization

The Fe/Cl ratios were measured with a Philips ESEM X230 scanning electron microscope equipped with an EDAX DX-4 microsonde. Elemental analyses (C, H, and N) were performed with a CE Instruments EA 1110 CHNS Elemental analyzer. Magnetic measurements were performed with a Quantum Design MPMS-XL-5 SQUID magnetometer with an applied magnetic field of 0.1 T. The solvated samples were deposited in the bottom of a glass tube covered with the mother liquor. This tube was used as the sample holder. For the solvent exchanged samples magnetic measurements, a previously desolvated polycrystalline sample was deposited in the bottom of a glass tube and kept in contact with a saturated atmosphere of the solvents for 24 h. After such time, the sample was quickly sealed and measured in the same tube used as a sample holder. The same sample was used for all the magnetic measurements of the solvent exchanged samples. Photomagnetic measurements were performed irradiating with a 30993 cylindrical Helium–Neon Laser system from Research Electro-Optics (red light, λ = 633 nm, optical power 12 mW cm^–2^) coupled via an optical fiber to the cavity of the SQUID magnetometer. It was verified that irradiation resulted in no significant change in the magnetic response due to heating of the sample. The photomagnetic samples consisted of a thin layer of compound whose weight was corrected by comparison of a thermal spin crossover curve with that of a more accurately weighted sample of the same compound. Solvated samples were protected with a grease immediately after being extracted from the mother liquor.

## Results and Discussion

### Synthesis

Slow liquid diffusion of diethyl ether in MeNO_2_ solutions of the complex afforded single crystals of [Fe(bpCOOEt_2_p)_2_](ClO_4_)_2_·1.5MeNO_2_ (**1**). If the slow diffusions in MeNO_2_ were left undisturbed for longer times, the crystals of **1** dissolved, and crystals of a new phase, [Fe(bpCOOEt_2_p)_2_](ClO_4_)_2_·MeNO_2_ (**2**) appeared. Finally, crystals of [Fe(bpCOOEt_2_p)_2_](ClO_4_)_2_·2MeNO_2_ (**3**) were obtained by vapor diffusion of diethyl ether in MeNO_2_. The purity of all these samples was checked by elemental analysis (see Experimental Section) and powder X-ray diffraction (PXRD). PXRD patterns of **1**, **2**, and **3** in contact with the mother liquor are consistent with the simulated ones obtained from the single-crystal X-ray diffraction structure (Figure S1). In contrast, PXRD patterns of filtered samples of **1** and **3** show important differences with respect to the simulated ones (see Figure S1), suggesting loss of crystallinity after removal of the mother liquor due to the disappearance of part of the lattice solvent molecules and replacement by water molecules as indicated by elemental analysis (see Experimental Section). Indeed, it was not possible to solve the structures by single-crystal X-ray diffraction above 260 K (**1**) and 280 K (**3**). On the contrary, PXRD patterns of **2** of the filtered and protected samples are very similar (Figure S1). This indicates that this phase is very stable against desolvation. Furthermore, it was possible to solve the single-crystal structures of the same crystal up to 400 K (desolvated phase) without losing the crystallinity. Motivated by these results, we carried out a study of resolvation of **2** in different solvents (Me_2_CO, MeNO_2_, CH_3_CN, and MeCOOH) by placing desolvated crystals of **2**, which were previously heated to 400 K, into a saturated atmosphere of those solvents for 24 h. This led to solvent-exchanged samples [Fe(bpCOOEt_2_p)_2_](ClO_4_)_2_·MeNO_2_ (**2·MeNO**_**2**_), [Fe(bpCOOEt_2_p)_2_](ClO_4_)_2_·MeCN (**2·MeCN**), [Fe(bpCOOEt_2_p)_2_](ClO_4_)_2_·0.5Me_2_CO (**2·Me**_**2**_**CO**), and [Fe(bpCOOEt_2_p)_2_](ClO_4_)_2_·MeCOOH (**2·MeCOOH**), which were structurally and magnetically characterized.

### Structure of **1**

**Figure 1:**
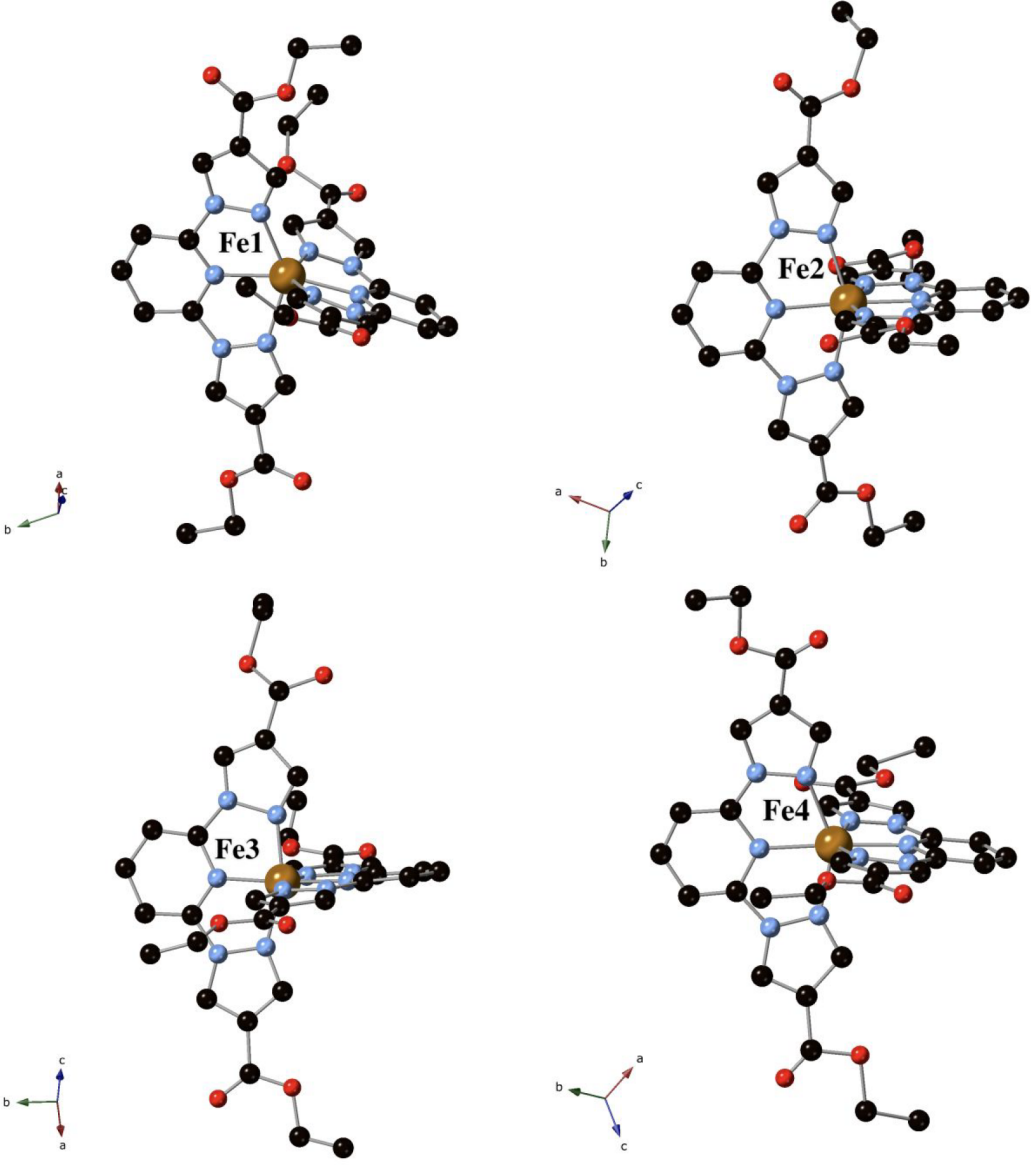
Crystallographically independent [Fe(bpCOOEt_2_p)_2_]^2+^ complexes in the structure of **1** at 120 K. Fe (orange), Cl (yellow), C (black), N (blue), and O (red). Hydrogen atoms have been omitted for clarity.

The crystal structure of **1** was solved at 120 K by single-crystal X-ray diffraction. **1** shows four crystallographically independent [Fe(bpCOOEt_2_p)_2_]^2+^ molecules with several disordered ethyl groups, seven perchlorate anions, and two MeNO_2_ molecules with occupancies of 0.5 (see Figure S2). The eighth perchlorate counterion and the remaining MeNO_2_ were highly disordered, and it was not possible to find them in the crystal structure. Nevertheless, EDX and elemental analysis are consistent with the presence of a 1:2 Fe/Cl ratio (see Experimental Section). The electron density map calculated by OLEX’s solvent mask command found 426.0 e^–^ in a void with a volume of 1685.1 Å^3^. This corresponds to two ClO_4_^–^ and 10 MeNO_2_ molecules per unit cell leading to the formula [Fe(bpCOOEt_2_p)_2_](ClO_4_)_2_·1.5MeNO_2_. The iron(II) ions present a distorted octahedral coordination geometry to the two tridentate bpCOOEt_2_p ligands similar to that of other Fe(II) bpp complexes. Fe–N distances and octahedral distortion parameters^32^ are consistent with one [Fe(bpCOOEt_2_p)_2_]^2+^ in the LS state ([Fe(bpCOOEt_2_p)_2_]^2+^ complex with Fe3), two in the HS state ([Fe(bpCOOEt_2_p)_2_]^2+^ complexes with Fe1 and Fe4), and one consisting of a mixture of HS and LS molecules ([Fe(bpCOOEt_2_p)_2_]^2+^ complex with Fe2) (see Tables 1 and 2). This is in agreement with the magnetic measurements (see below). They present short contacts involving CH groups of pyridine or pyrazole rings with the CO, CH_2_, and CH_3_ groups of the carboxyethyl groups. The ethoxy groups of one of the two bpCOOEt_2_p ligands of [Fe(bpCOOEt_2_p)_2_]^2+^ complexes with Fe1 and Fe3 point to opposite directions, thus exhibiting a syn,anti configuration. In the second one, they point to the same directions leading to a syn,syn configuration. In [Fe(bpCOOEt_2_p)_2_]^2+^ complexes with Fe2, syn,syn configurations of the ethoxy groups are observed in the two bpCOOEt_2_p ligands. Finally, syn,anti configurations are observed in the two bpCOOEt_2_p ligands in [Fe(bpCOOEt_2_p)_2_]^2+^ complexes with Fe4 (see Figure 1).

**Table 1:** Fe–N Distances (Å) in the [Fe(bpCOOEt_2_p)_2_]^2+^ Complexes of **1** at 120 K

Fe1–N1	2.162(9)	Fe3–N21	1.972(10)
Fe1–N3	2.140(8)	Fe3–N23	1.913(8)
Fe1–N5	2.166(9)	Fe3–N25	2.017(10)
Fe1–N6	2.151(9)	Fe3–N26	2.004(10)
Fe1–N8	2.132(7)	Fe3–N28	1.937(8)
Fe1–N10	2.129(9)	Fe3–N30	1.996(10)
Fe2–N11	2.053(11)	Fe4–N31	2.107(8)
Fe2–N13	2.044(9)	Fe4–N33	2.085(7)
Fe2–N15	2.062(10)	Fe4–N35	2.132(7)
Fe2–N16	2.066(10)	Fe4–N36	2.126(8)
Fe2–N18	2.051(9)	Fe4–N38	2.089(8)
Fe2–N20	2.046(9)	Fe4–N40	2.165(9)

**Table 2:** Σ and Θ Distortion Octahedral Parameters in the [Fe(bpCOOEt_2_p)_2_]^2+^ Complexes of **1**, **2**, and **3**

compound	iron	*T* (K)	Σ (deg)	Θ (deg)
**1**	Fe1	120	167.6(13)	553(3)
**1**	Fe2	120	121.9(16)	400(3)
**1**	Fe3	120	91.8(13)	303(4)
**1**	Fe4	120	149.4(14)	475(3)
**2**	Fe1	120	92.9(8)	296.7(17)
**2**	Fe1	300	113.3(9)	366(2)
**2**	Fe1	400	135.0(14)	443(3)
**2**	Fe1	120^a^	92.4(4)	281.1(10)
**3**	Fe1	150	160.3(3)	531.6(8)
**3**	Fe2	150	85.7(2)	281.7(8)
**3**	Fe1	280	159.8(4)	534.5(9)
**3**	Fe2	280	87.2(2)	286.7(8)

a After being heated to 400 K.

### Structure of [Fe(bpCOOEt_2_p)_2_](ClO_4_)_2_·MeNO_2_ (**2**)

**Figure 2:**
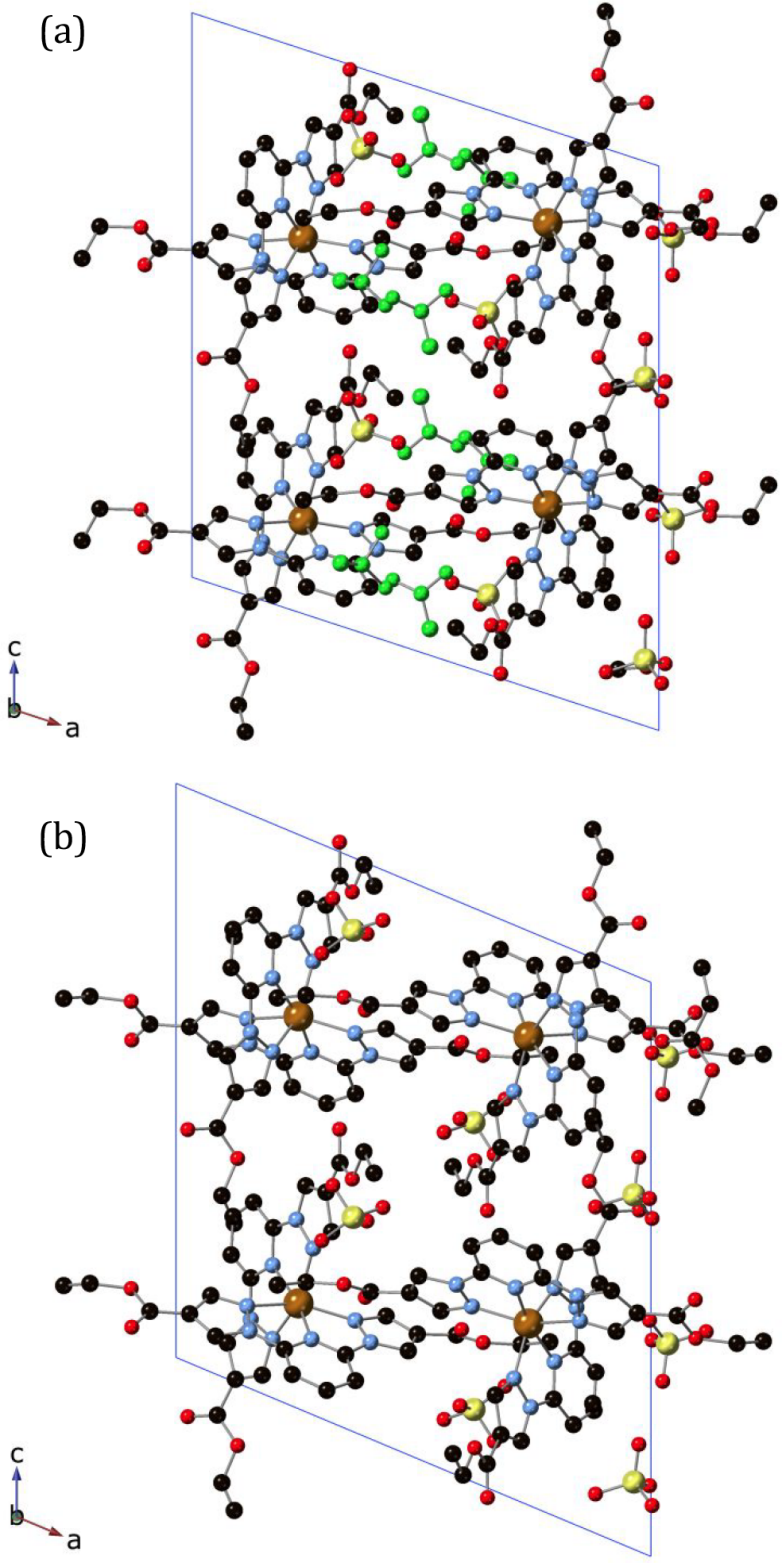
Projection of the structure of **2** at 120 K (a) and 400 K (b) in the *ac* plane. Fe (orange), Cl (yellow), C (black), N (blue), and O (red). MeNO_2_ molecules in the structure at 120 K have been colored in green. Hydrogen atoms have been omitted for clarity.

The crystal structure of **2** was solved by single-crystal X-ray diffraction at 120, 300, and 400 K. At all temperatures, it crystallizes in a monoclinic crystal system with a centrosymmetric *P*2_1_/*c* space group. The asymmetric unit at 120 K is composed of one [Fe(bpCOOEt_2_p)_2_]^2+^ cation, two ClO_4_^–^ anions (one disordered at all temperatures), and two MeNO_2_ molecules with an occupancy of 0.5 (see Figure 2). Although it was not possible to find the MeNO_2_ solvent molecules in the structure at 300 K due to a high degree of disorder, the electron density map calculated by OLEX’s solvent mask command found 59.5 e^–^ in two voids each with a volume of 278.5 Å^3^. This is consistent with the presence of one MeNO_2_ molecule in the asymmetric unit cell. Therefore, this compound can be formulated as [Fe(bpCOOEt_2_p)_2_](ClO_4_)_2_·MeNO_2_ (**2**) at 120 and 300 K. However, elemental analysis at 300 K of the filtered samples is more consistent with a half molecule of MeNO_2_ and three water molecules, suggesting a partial loss of the MeNO_2_ solvent molecule and replacement with water molecules after extracting the crystals from the mother liquor for longer times (see the Experimental section). The asymmetric unit at 400 K is composed by only one [Fe(bpCOOEt_2_p)_2_]^2+^ cation and two ClO_4_^–^ anions leading to the formula [Fe(bpCOOEt_2_p)_2_](ClO_4_)_2_ (see Figure 2). The loss of the solvent molecules upon increasing the temperature is followed by a decrease in the unit cell volume as expected (4587.9(13) at 300 K to 4545.4(15) Å^3^ at 400 K, see Table S1). The structure of the same desolvated crystal was solved again at 120 K after being heated to 400 K confirming that the crystallinity is not lost after desolvation.

The Fe–N bond lengths and octahedral distortion parameters at 120 K (1.913(4)–1.984(4) Å) and 300 K (1.968(5)–2.061(5) Å) indicate that the complex undergoes a gradual spin transition with temperature from 120 to 300 K as observed in the magnetic measurements (see Tables 2 and 3). On the other hand, Fe–N distances of the desolvated crystal at 400 K (2.061(8)–2.151(8) Å) and 120 K (1.896(3)–1.998(3) Å) also agree with magnetic measurements suggesting that both solvated and desolvated sample exhibit similar gradual spin transitions. The ethoxy groups of the two bpCOOEt_2_p ligands of each complex exhibit syn,anti configurations in all these structures. Neighboring [Fe(1bpCOOEt_2_p)_2_]^2+^ cations present CO···π and CH···π interactions between the carbonyl or ethyl group and the pyrazolyl and pyridine rings plus numerous contacts involving ethyl groups, carbonyl and CH groups with pyrazole or pyridine rings and O atom from ethoxy group with CH_3_ from ethyl or CH from pyridine ring. This gives rise to a complicate network of intermolecular interactions that form channels along the *b* axis, which are occupied by MeNO_2_ solvent molecules and ClO_4_^–^ counteranions (see Figure 2). Interestingly, this network of interactions is maintained after desolvation of the compound and channels running along the *b* axis are observed in the structure of the desolvated compound at 120 and 400 K, containing only ClO_4_^–^ counteranions (see Figure 2 and Figures S3 and S4 and associated text in the Supporting Information). The location of part of the MeNO_2_ solvent molecules in the parallel channels along the *b* axis could enable a pathway for their ordered diffusion in or out the lattice. This could explain the persistence of the crystallinity after desolvation. In addition, these channels could be used to resolvate the compound with MeNO_2_ and other solvents in a SCSC fashion as described below.

**Table 3:** Fe–N Distances (Å) in the [Fe(bpCOOEt_2_p)_2_]^2+^ Complexes of **2**

120 K	Fe1–N1	1.968(5)	400 K	Fe1–N1	2.091(9)
	Fe1–N3	1.923(4)		Fe1–N3	2.076(7)
	Fe1–N5	1.983(5)		Fe1–N5	2.151(8)
	Fe1–N6	1.984(4)		Fe1–N6	2.103(9)
	Fe1–N8	1.913(4)		Fe1–N8	2.061(8)
	Fe1–N10	1.999(5)		Fe1–N10	2.118(9)
300 K	Fe1–N1	2.046(6)	120 K^a^	Fe1–N1	1.998(3)
	Fe1–N3	1.980(5)		Fe1–N3	1.896(3)
	Fe1–N5	2.061(5)		Fe1–N5	1.969(3)
	Fe1–N6	2.028(6)		Fe1–N6	1.968(3)
	Fe1–N8	1.968(5)		Fe1–N8	1.899(3)
	Fe1–N10	2.035(6)		Fe1–N10	1.975(3)

a After being heated to 400 K.

### Structure of the Solvent-Exchanged Samples [Fe(bpCOOEt_2_p)_2_](ClO_4_)_2_·MeNO_2_ (**2·MeNO_2_**), [Fe(bpCOOEt_2_p)_2_](ClO_4_)_2_·MeCN (**2·MeCN**), [Fe(bpCOOEt_2_p)_2_](ClO_4_)_2_·0.5Me_2_CO (**2·Me_2_CO**), and [Fe(bpCOOEt_2_p)_2_](ClO_4_)_2_·MeCOOH (**2·MeCOOH**)

**Figure 3:**
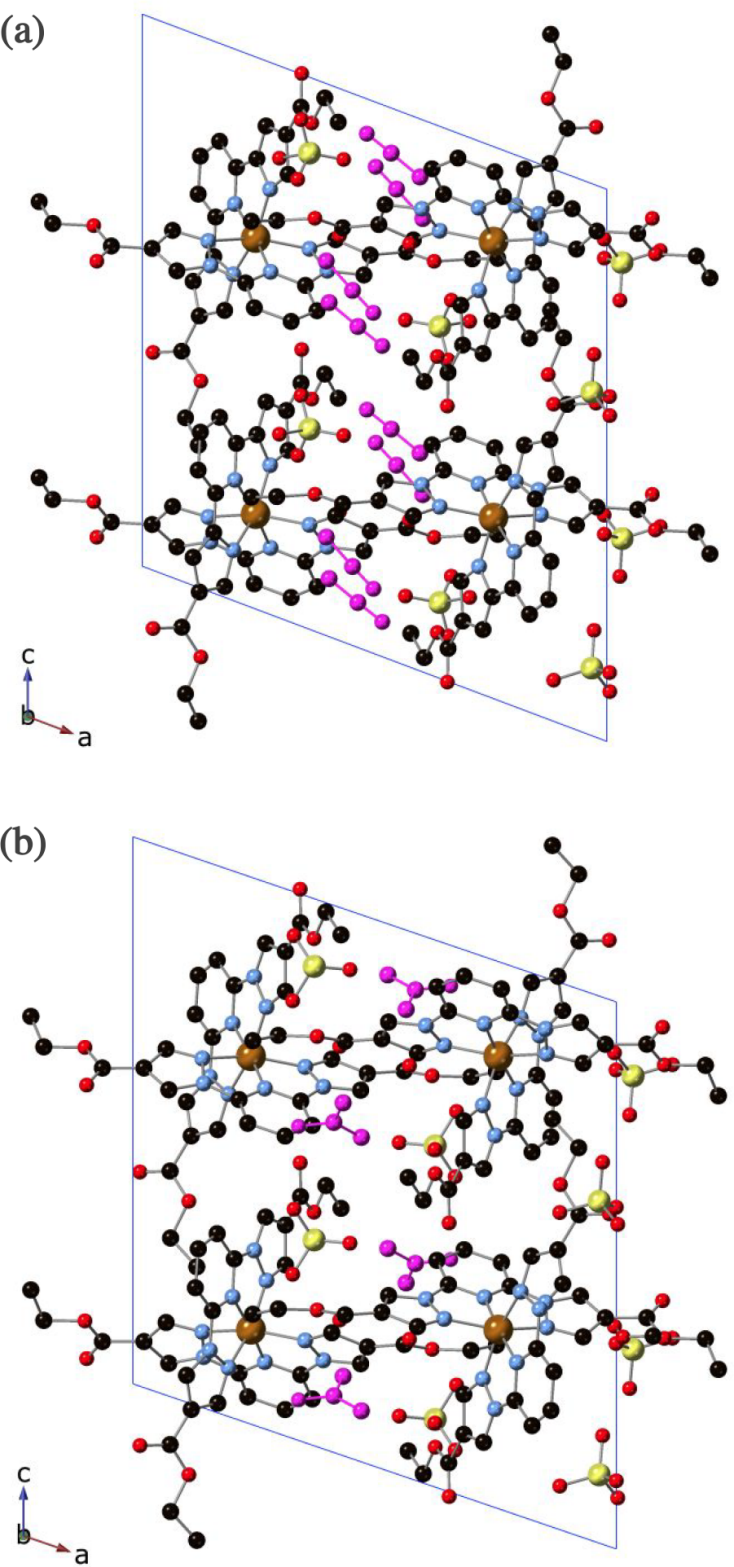
Projection of the structure of **2·MeCN** (a) and **2·Me**_**2**_**CO** (b) at 120 K in the *ac* plane. Fe (orange), Cl (yellow), C (black), N (blue), and O (red). MeCN and Me_2_CO solvent molecules have been colored in violet. Hydrogen atoms have been omitted for clarity.

Single-crystal X-ray diffraction measurements performed in crystals of **2** previously desolvated at 400 K and exposed to vapors of different solvents confirmed the obtention of analogous structures with the insertion of the solvent molecules in the above-mentioned cavities (see Table S1 and Figures 3 and S5). Thus, the structure of **2·Me**_**2**_**CO** shows one disordered Me_2_CO molecule, which has been solved with two possible configurations with 25% of occupancy, while in the structures of **2·MeCN** and **2·MeCOOH**, one MeCN or MeCOOH molecule entered in the structure, solved as two MeCN or MeCOOH molecules with an occupancy of 0.5. Finally, the resolvated **2·MeNO**_**2**_ sample recovers the initial MeNO_2_ content with a different disorder (three crystallographically independent MeNO_2_ molecules with occupancies of 0.5, 0.25 and 0.25). To study the effect of resolvation in the spin state of the Fe(II) complexes we have measured the unit cell volume (see Table S1) and Fe–N distances at 120 K. In all cases, typical LS values are obtained (1.927(4)–2.024(5) Å for **2·MeCN**, 1.906(4)–2.008(5) Å for **2·MeNO**_**2**_, 1.909(3)–2.003(4) Å for **2·MeCOOH**, and 1.890(4)-2.012(4) Å for **2·Me**_**2**_**CO**). Furthermore, an increase of unit cell volume (4498.6(3) Å^3^ for **2·MeCN**, 4403.7(6) Å^3^ for **2·MeNO**_**2**_, 4372.87(19) Å^3^ for **2·MeCOOH**, and 4303.9(14) Å^3^ for **2·Me**_**2**_**CO**) compared with that of the desolvated compound at 120 K (4246.7(3) Å^3^) is obtained. The smaller Fe–N distances and unit cell volume found in **2·Me**_**2**_**CO** indicate that the LS state is more favored in this compound. In contrast, the greater values of these two parameters in **2·MeCN** are characteristic of a higher HS fraction. This is further confirmed by magnetic measurements (see below). These results suggest that it is possible to tune the spin state of this compound by resolvation with different vapors.

### Structure of [Fe(bpCOOEt_2_p)_2_](ClO_4_)_2_·2MeNO_2_ (**3**)

**Figure 4:**
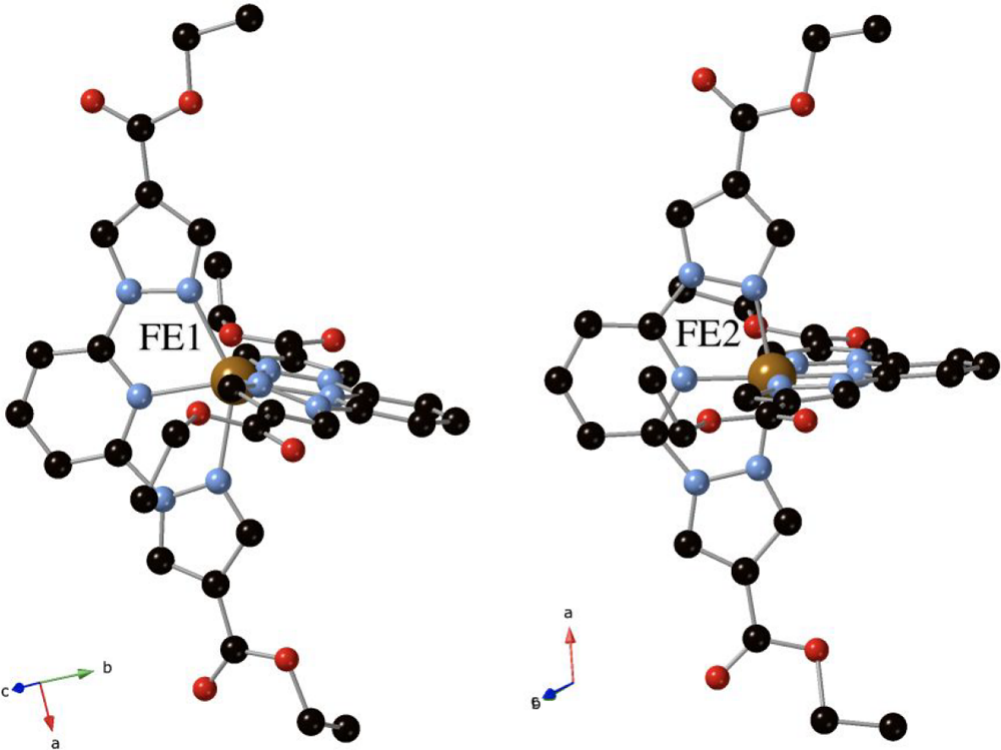
Crystallographically independent [Fe(bpCOOEt_2_p)_2_]^2+^ complexes in the structure of **3** at 150 K. Fe (orange), Cl (yellow), C (black), N (blue), and O (red). Hydrogen atoms have been omitted for clarity.

The structure of **3** was solved by single-crystal X-ray diffraction at 150 and 280 K in the centrosymmetric *P*1̅ space group. At higher temperatures, the structure could not be solved due to the loss of crystallinity. The asymmetric unit contains two [Fe(bpCOOEt_2_p)_2_]^2+^ cations, four ClO_4_^–^ anions, and four MeNO_2_ molecules with some disorder (see Figure S6). The structure shows the following formula [Fe^II^(bpCOOEt_2_p)_2_](ClO_4_)_2_·2MeNO_2_ at 150 K, while elemental analysis at 300 K is more consistent with replacement of part of the MeNO_2_ molecules by water molecules that explains the differences observed in PXRD. It was not possible to find MeNO_2_ solvent molecules in the structure at 280 K due to severe disorder. It was removed from the electron density map using the OLEX solvent mask command. One void of 788.7 Å^3^ was found in the unit cell occupied by approximately six MeNO_2_ molecules (194.3 e^–^). Thus, the structure at 280 K is consistent with the following formula [Fe^II^(bpCOOEt_2_p)_2_](ClO_4_)_2_·1.5MeNO_2_. Therefore, there is a decrease in the number of MeNO_2_ in the structure after heating from 150 to 280 K in the diffractometer. This loss of solvent molecules is not accompanied by a decrease in unit cell volume (4709.6(2) at 120 K to 4839.3(2) Å^3^ at 280 K). The structure contains two crystallographically independent Fe(II) complexes (see Figure 4). One of them with typical HS Fe–N bond distances (2.131(2)–2.202(2) Å at 150 K and 2.138(2)–2.214(3) Å at 280 K for [Fe^II^(bpCOOEt_2_p)_2_]^2+^ complex with Fe1) and the other one with typical LS ones (1.8993(19)–1.982(2) Å at 150 K and 1.907(2)–1.989(2) Å at 280 K for [Fe^II^(bpCOOEt_2_p)_2_]^2+^ complex with Fe2). The Fe–N bond distances and octahedral distortion parameters at 150 K are consistent with the 50% of HS/LS state found in the magnetic properties of the sample measured in contact with the mother liquor (see below and Tables 2 and 4). However, the very similar Fe–N distances and octahedral distortion parameters at 120 and 280 K do not agree with the clear increase of the HS fraction at this temperature range observed in the magnetic properties (see below). This could be a consequence of the partial desolvation observed by single-crystal X-ray diffraction, which is not taking place in the sample used for the magnetic measurements since it was protected with the mother liquor. The ethoxy groups of the four bpCOOEt_2_p ligands of these two complexes exhibit syn,syn configurations (see Figure 4). Finally, the complexes display a complicate network of intermolecular interactions involving CH_2_ and CH_3_ groups of the ethyl groups, CO groups, and pyridine and pyrazole rings.

**Table 4:** Fe–N Distances (Å) in the [Fe(bpCOOEt_2_p)_2_]^2+^ Complexes of **3**

150 K	Fe1–N1	2.202(2)	280 K	Fe1–N1	2.173(2)
	Fe1–N3	2.151(2)		Fe1–N3	2.138(2)
	Fe1–N5	2.182(2)		Fe1–N5	2.150(2)
	Fe1–N6	2.139(2)		Fe1–N6	2.214(3)
	Fe1–N8	2.131(2)		Fe1–N8	2.160(2)
	Fe1–N10	2.166(2)		Fe1–N10	2.183(2)
	Fe2–N11	1.973(2)		Fe2–N11	1.981(2)
	Fe2–N13	1.9016(19)		Fe2–N13	1.907(2)
	Fe2–N15	1.980(2)		Fe2–N15	1.989(2)
	Fe2–N16	1.972(2)		Fe2–N16	1.978(2)
	Fe2–N18	1.8993(19)		Fe2–N18	1.907(2)
	Fe2–N20	1.982(2)		Fe2–N20	1.984(2)

### Magnetic Properties of **1**, **2**, and **3**

**Figure 5:**
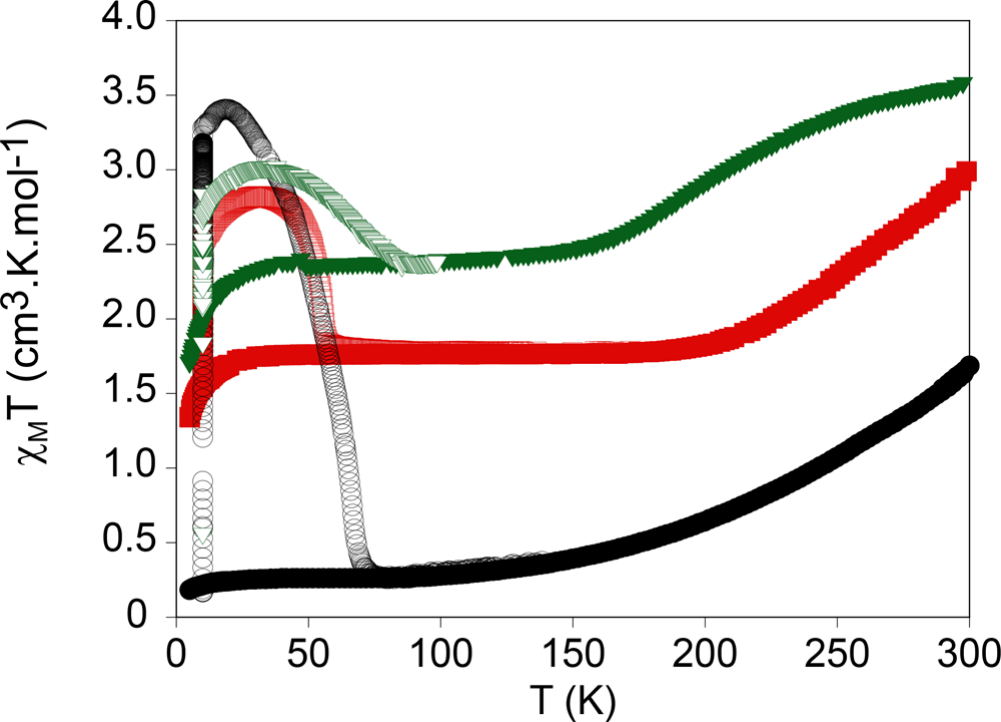
Thermal variation of χ_M_*T* for **1** (green triangles), **2** (black circles), and **3** (red squares). Full triangles, circles, or squares: data recorded without irradiation; empty triangles, circles, or squares: data recorded after irradiation at 10 K.

Magnetic properties of the three solvatomorphs show a variety of magnetic behaviors (see Figure 5). For instance, the molar magnetic susceptibility times temperature (χ_M_*T*) of **1** presents a value of 2.4–2.5 cm^3^·K·mol^–1^ in the temperature range 40–150 K, which corresponds to ∼2/3 of the Fe(II) centers with HS configuration taking 3.5 cm^3^·K·mol^–1^ as a reference of the full HS state. This is consistent with the four crystallographically independent Fe(II) complexes (two in the HS state, one in the LS state, and one with a mixture of LS and HS molecules as indicated by the Fe–N distances from the molecular structure at 120 K; see Table 1). A decrease in χ_M_*T* value is found below 30 K due to the zero-field splitting. At temperatures above 150 K, χ_M_*T* shows a gradual increase to reach a value close to 3.6 cm^3^·K·mol^–1^ typical for all the iron centers in a HS configuration. Therefore, from 150 to 300 K, **1** displays a complete and reversible spin transition of two of the four crystallographically independent iron centers present in the asymmetric unit cell. The partially desolvated sample obtained by filtering the compound shows a similar behavior with lower χ_M_*T* values below 150 K and a more gradual and incomplete spin transition at higher temperatures (see Figure S7). The magnetic response of **2** displays a value below 0.5 cm^3^·K·mol^–1^ in the 2–150 K temperature range. Upon increasing the temperature, a gradual thermal spin transition is observed to reach a value of 3.1 cm^3^·K·mol^–1^ at 400 K. Desolvation of the compound does not seem to change the magnetic behavior as thermal variation of χ_M_*T* presents small changes after heating at 400 K or measuring in contact with the mother liquor (see Figure S8). Therefore, a gradual and almost complete spin transition takes place in the 150–400 K temperature range for the solvated and desolvated compounds with a thermal SCO temperature *T*_1/2_ (*T*_1/2_ = temperature of 50% HS to LS conversion) of ∼300 K. These results are in agreement with the Fe–N bond distances obtained from single-crystal structures at different temperatures. Finally, χ_M_*T* of **3** in contact with the mother liquor displays a constant value of ca. 1.7 cm^3^·K·mol^–1^ below 200 K. This value is consistent with half of the iron centers in the HS state and agrees with the Fe–N bond distances observed in single-crystal diffraction at 120 K. Upon increasing the temperature, a gradual spin transition is observed until reaching a value close to 3.2 cm^3^·K·mol^–1^ at 300 K, which corresponds to almost a full conversion of the iron ions to the HS state. This behavior is reversible upon cooling. Therefore, from 150 to 300 K, **3** displays an almost complete and reversible spin transition of one of the two crystallographically independent iron centers. This SCO behavior is lost in the filtered sample (see Figure S9), which shows a constant value of 2.1–2.3 cm^3^·K·mol^–1^ in the 50–280 K temperature range, in agreement with the crystal structure at 280 K (see above).

The three compounds were protected with an oil, and their response with light was studied (see Figure 5). They show a clear increase of the χ_M_*T* value after irradiating with 660 nm and, hence, LIESST effect. Maximum values of 3.0 cm^3^·K·mol^–1^ (**1**), 3.4 cm^3^·K·mol^–1^ (**2**), and 2.8 cm^3^·K·mol^–1^ (**3**) were reached after switching down the light. This increase is consistent with a partial spin transition of the iron centers in the LS at 10 K to the HS state, which is almost complete in the case of **2**. Increasing the temperature gives rise to a gradual thermal relaxation obtaining values consistent with those before irradiation. The *T*(LIESST) values of the three compounds, defined as the minimum of the derivate of χ_M_*T* after irradiation with temperature, are 69 K (**1**), 67 K (**2**), and 56 K (**3**). The *T*(LIESST) value of **2** is consistent with the linear correlation between *T*_1/2_ and the *T*(LIESST) found for this family of compounds and described by the formula *T*(LIESST) = *T*_0_ – 0.3*T*_1/2_ (*T*_0_ = 150 K).^33−36^

### Magnetic Properties of the Solvent-Exchanged Samples **2·MeCN**, **2·Me_2_CO**, and **2·MeCOOH**

**Figure 6:**
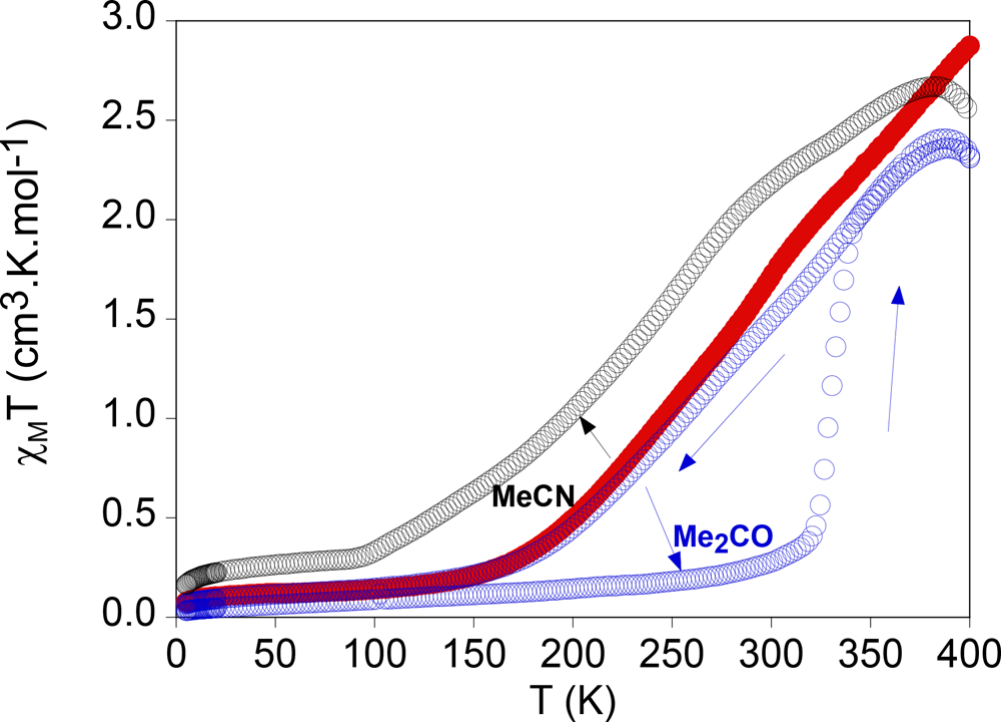
Thermal variation of χ_M_*T* for desolvated **2** (red full circles), **2·MeCN** (empty black circles), and **2·Me**_**2**_**CO** (empty blue circles).

Magnetic properties of the resolvated compounds were measured in the same sample, which was heated to 400 K and then placed in a saturated atmosphere of the solvent of interest for 24 h. The same sample was then heated to 400 K, and the same treatment was repeated with other solvent. **2·Me**_**2**_**CO** exhibits the most drastic change in the magnetic properties with respect to desolvated **2** of all these samples (see Figure 6). Thus, the χ_M_*T* value of **2·Me**_**2**_**CO** is close to 0 cm^3^·K·mol^–1^ from 5 to 300 K. This is consistent with the LS state and agrees with the decrease in the Fe–N bond distances observed by single-crystal X-ray diffraction experiments (see above). At a higher temperatures, there is an abrupt increase of χ_M_*T* to reach 2.1 cm^3^·K·mol^–1^ at 350 K (Figure 6). This change of spin state is associated with the loss of the Me_2_CO solvent molecules. Indeed, χ_M_*T* values above 350 K agree with those of the desolvated sample. Interestingly, the gradual spin transition of the desolvated sample is recovered in the cooling cycle from 400 to 5 K, which confirms the reversibility of the desolvation/resolvation process (Figure 6). On the other hand, the insertion of MeCN slightly stabilizes the HS state. Thus, **2·MeCN** shows a gradual spin transition with higher χ_M_*T* values than those found in desolvated **2** at all temperatures. Therefore, the subtle increase in Fe–N bond distances in **2·MeCN** at 120 K (see above) is explained by the small fraction of molecules in the HS state at this temperature. Magnetic properties of **2·MeCOOH** are very similar to those of the desolvated sample (see Figure S10). Although it is difficult to rationalize this behavior because it depends on many factors, several trends are observed. On the one hand, the unit cell volume of **2·MeCN** (4498.6(3) Å^3^) is much higher than that of **2·Me**_**2**_**CO** (4303.9(14) Å^3^). Therefore, the larger chemical pressure induced by the decrease in unit cell volume in **2·Me**_**2**_**CO** could stabilize the LS state as observed. On the other hand, removal of the MeNO_2_ solvent molecule and replacement by MeCN or Me_2_CO could change the intermolecular interactions with the [Fe^II^(bpCOOEt_2_p)_2_]^2+^ complexes. This could change the crystal field around iron(II), taking into account that the spin state of bpp iron(II) complexes is very sensitive to intermolecular interactions with solvent molecules or counteranions.^19−22,37,38^ In this sense, the MeCN, Me_2_CO, and MeNO_2_ solvent molecules in these structures present numerous short contacts with the pyrazole and pyridine rings of the [Fe^II^(bpCOOEt_2_p)_2_]^2+^ complexes, and these interactions could play a role in the observed tuning of the spin state.

## Conclusions

The presence of ethylcarboxy groups in the pyrazole ring of bpp with the capability to rotate around the C–C bond and adopt different conformations could be at the origin of the obtention of three different solvatomorphs of the Fe(II) complex of this ligand in MeNO_2_. In all of them different combinations of the syn/anti or syn/syn conformations of the ethoxy groups are obtained. These compounds present SCO properties ranging from partial to complete thermal spin transitions and an LIESST effect. This contrast to previous results with CF_3_CH_2_OH solvate, in which spin transition was not observed. The presence of ethyl groups prevents the formation of strong intermolecular interactions such as π–π bonding interactions between the pyrazole and pyridine rings, which are typically observed in bpp complexes, leading in all cases to gradual spin transitions with temperature. In solvatomorph **2**, the flexibility afforded by this ethylcarboxy groups together with a robust network of intermolecular interactions is crucial in the formation of a stable structure with channels that can be reversibly filled with different solvent molecules. This allows the tuning of the SCO properties of the compound. In contrast to previous examples of molecular-based SCO compounds displaying solvent-sensitive SCO properties, the complexes are not linked through strong intermolecular interactions such as π–π stacking or hydrogen-bonding. We conclude then that the use of flexible groups in the 4-pyrazolyl substituents of bpp ligand is a suitable strategy for the preparation of robust structures where the incorporation and diffusion of different solvent molecules is feasible and completely reversible. This has been tested with a few solvents but could be extended to other solvents or gas molecules. The cooperativity and abruptness of the spin transitions could be improved by combining these flexible substituents with other ones enabling stronger intermolecular interactions such as carboxylic acid or unsubstituted pyrazolyl units. This work is in progress.

## Supporting Information Available

Structural tables, views of the structures, powder X-ray diffraction patterns of the compounds, and magnetic measurements (PDF)
