# Supplementary material for: Iron(II) Complexes of 2,6-Di[4-(ethylcarboxy)pyrazol-1-yl]pyridine with Reversible Guest-Modulated Spin-Crossover Behavior
Source: Cryst Growth Des. 2023 Mar 2;23(4):2730–8. doi: 10.1021/acs.cgd.2c01524 (PMC10080648; doi:10.1021/acs.cgd.2c01524)
Supplement: Supplementary file 1 — cg2c01524_si_001.pdf [file cg2c01524_si_001.pdf]

SUPPORTING INFORMATION

Iron(II) Complexes of 2,6-di[4-(ethylcarboxy)pyrazol-1-yl]pyridine with Reversible Guest-modulated Spin-Crossover Behavior

Víctor García-López,[a] *Hanane El Mansour El Jastimi,*[a] *Jana Juráková,*[a,b] *Miguel Clemente-León*[a*] *and Eugenio Coronado*[a]

[a]Instituto de Ciencia Molecular (ICMol), Universidad de Valencia, Catedrático José Beltrán 2, 46980 Paterna, Spain

[b]Central European Institute of Technology, Brno University of Technology, Purkyňova 123, 61200 Brno, Czech Republic

CONTENTS

Experimental details of the structure of **2.**

Table S1. Crystallographic data for **1**, **2** and **3** and solvent exchanged samples.

Figure S1. Experimental and simulated powder X-ray diffraction (PXRD) patterns of **1** (a), **2** (b) and **3** (c) in contact with the mother liquor and filtered.

Figure S2. Projection of the structure of **1** at 120 K in the ac plane. Fe (orange), Cl (yellow), C (black), N (blue) and O (red). Hydrogen atoms have been omitted for clarity.

Figure S3. Intermolecular interactions between $[Fe(1bpCOOEt_2p)_2]^{2+}$ complexes of **2** at 400 K.

Figure S4. Intermolecular interactions between $[Fe(1bpCOOEt_2p)_2]^{2+}$ complexes of **2** at 400 K.

Figure S5. Projection of the structure of **2·MeNO$_2$ (a)** and **2·Me$_2$CO (b)** at 120 k in the *ac* plane.

Figure S6. Projection of the structure of **3** at 120 K in the *bc* plane. Fe (orange), Cl (yellow), C (black), N (blue) and O (red). Hydrogen and disordered atoms have been omitted for clarity.

Figure S7. Thermal variation of $\chi_M T$ for **1**.

Figure S8. Thermal variation of $\chi_M T$ for **2**.

Figure S9. Thermal variation of $\chi_M T$ for **3**

Figure S10. Thermal variation of $\chi_M T$ for **2·MeCOOH**.

Experimental details of the structure of 2.

Although it was not possible to solve the $MeNO_2$ solvent molecules in the structure at 300 K due to a high degree of disorder, the electron density map calculated by OLEX's solvent mask command found 59.5 e- in two voids each with a volume of 278.5 $Å^3$. This is consistent with the presence of four $MeNO_2$ molecules in the unit cell, therefore, two half MeNO2 molecules in asymmetric unit cell. Elemental analysis at 300 K is more consistent with half molecule of nitromethane and three water molecules, suggesting a partial loss of the nitromethane solvent molecule and replacement with water molecules after extracting the crystals from the mother liquor, or the coexistence of crystals with a different degree of desolvation (see the Experimental section). At 120 K one ethyl group of each bppCOOEt ligand is disordered and was solved with two possible configurations for the terminal methyl group with a 0.5 occupancy. At higher temperatures, 300 and 400 K, the degree of disorder increases and now an additional ethyl group is also disordered which has been solved with two different configurations with a 50% occupancy.

Table S1. Crystallographic data for **1**, **2** and **3** and solvent exchanged samples.

Compound	**1**	**3**	**3**
Empirical formula	$C_{137}H_{140}Cl_7Fe_4N_{42}O_{64}$	$C_{72}H_{80}Cl_4Fe_2N_{24}O_{40}$	$C_{68}H_{65}Cl_4Fe_2N_{20}O_{32}$
Formula weight	3870.45	2175.10	1927.90
Temperature/K	120.00(10)	151(2)	279.95(10)
Crystal system	triclinic	triclinic	triclinic
Space group	P-1	P-1	P-1
a/Å	20.7328(4)	16.0844(4)	16.1467(4)
b/Å	22.4930(6)	18.4255(5)	18.6139(5)
c/Å	24.3008(5)	18.5004(4)	18.7148(4)
α/°	105.781(2)	106.799(2)	107.319(2)
β/°	107.909(2)	113.217(2)	96.071(2)
γ/°	103.245(2)	94.381(2)	111.700(2)
Volume/Å^3	9752.9(4)	4709.6(2)	4839.3(2)
Z	2	2	2
ϱ_{calc}g/cm^3	1.319	1.534	1.323
μ/mm^{-1}	0.477	0.524	0.494
F(000)	3978.0	2240.0	1984.0
Crystal size/mm^3	0.25 × 0.25 × 0.08	0.6 × 0.5 × 0.5	0.6 × 0.5 × 0.5
Radiation	MoKα (λ = 0.71073)	MoKα (λ = 0.71073)	MoKα (λ = 0.71073)
2Θ range for data collection/°	5.648 to 52.744	5.73 to 59.736	5.744 to 59.792
Index ranges	-27 ≤ h ≤ 27, -31 ≤ k ≤ 31, -32 ≤ l ≤ 33	-22 ≤ h ≤ 21, -25 ≤ k ≤ 25, -25 ≤ l ≤ 25	-21 ≤ h ≤ 22, -25 ≤ k ≤ 25, -26 ≤ l ≤ 26
Reflections collected	141434	90583	92390
Independent reflections	39857 [R_{int} = 0.1256, R_{sigma} = 0.3264]	24570 [R_{int} = 0.0289, R_{sigma} = 0.0322]	25236 [R_{int} = 0.0425, R_{sigma} = 0.0488]
Data/restraints/parameters	39857/228/2312	24570/39/1371	25236/139/1255
Goodness-of-fit on F^2	0.983	1.028	1.041
Final R indexes [I>=2σ (I)]	R_1 = 0.1244, wR_2 = 0.3263	R_1 = 0.0589, wR_2 = 0.1558	R_1 = 0.0610, wR_2 = 0.1549

aR1(F) = ∑||Fo|–|Fc||/∑|Fo|; bwR2(F2) = [∑w(Fo2–Fc2)2/∑wFo4]½; cS(F2) = [∑w(Fo2–Fc2)2/∑n + r – p)]½

Compound	**2**	**2**	**2**	**2** (after being heated to 400 K
Empirical formula	$C_{35}H_{37}Cl_2FeN_{11}O_{18}$	$C_{34}H_{34}Cl_2FeN_{10}O_{16}$	$C_{34}H_{34}Cl_2FeN_{10}O_{16}$	$C_{34}H_{34}Cl_2FeN_{10}O_{16}$
Formula weight	1026.50	965.46	965.46	965.46
Temperature/K	120.05(10)	300(2)	399.95(10)	120.2(7)
Crystal system	monoclinic	monoclinic	monoclinic	monoclinic
Space group	$P2_1/c$	$P2_1/c$	$P2_1/c$	$P2_1/c$
a/Å	17.3428(8)	17.751(3)	17.962(3)	17.9860(7)
b/Å	13.7324(5)	13.6581(18)	13.6805(17)	13.0539(4)
c/Å	19.9243(10)	20.059(4)	20.060(4)	19.1425(9)
α/°	90	90	90	90
β/°	108.185(5)	109.369(18)	112.76(2)	109.113(5)
γ/°	90	90	90	90
Volume/Å^3	4508.1(4)	4587.9(13)	4545.4(15)	4246.7(3)
Z	4	4	4	4
ϱ_{calc}g/cm^3	1.512	1.395	1.411	1.510
μ/mm^{-1}	0.539	0.521	0.526	0.563
F(000)	2112.0	1976.0	1984.0	1984.0
Crystal size/mm^3	0.5 × 0.1 × 0.05	0.5 × 0.1 × 0.05	0.5 × 0.1 × 0.05	0.5 × 0.1 × 0.05
Radiation	MoKα (λ = 0.71073)	MoKα (λ = 0.71073)	MoKα (λ = 0.71073)	MoKα (λ = 0.71073)
2Θ range for data collection/°	5.766 to 59.904	6.342 to 52.744	5.75 to 50.7	5.72 to 59.868
Index ranges	-22 ≤ h ≤ 23, -19 ≤ k ≤ 17, -26 ≤ l ≤ 26	-24 ≤ h ≤ 22, -19 ≤ k ≤ 17, -26 ≤ l ≤ 26	-21 ≤ h ≤ 22, -17 ≤ k ≤ 17, -24 ≤ l ≤ 25	-23 ≤ h ≤ 24, -18 ≤ k ≤ 17, -25 ≤ l ≤ 25
Reflections collected	40030	34144	31638	37789
Independent reflections	11852 [Rint = 0.0841, Rsigma = 0.1296]	9360 [R_{int} = 0.1423, R_{sigma} = 0.1886]	8321 [R_{int} = 0.1746, R_{sigma} = 0.2004]	11136 [R_{int} = 0.0729, R_{sigma} = 0.1001]
Data/restraints/ parameters	11852/16/671	9360/99/645	8321/110/636	11136/0/572
Goodness-of-fit on F^2	1.063	0.913	0.894	1.066
Final R indexes [I>=2σ (I)]	R1 = 0.0947, wR2 = 0.2393	R_1 = 0.0792, wR_2 = 0.1603	R_1 = 0.1030, wR_2 = 0.2574	R_1 = 0.0599, wR_2 = 0.1210

aR1(F) = ∑||Fo|–|Fc||/∑|Fo|; bwR2(F2) = [∑w(Fo2–Fc2)2/∑wFo4]½; cS(F2) = [∑w(Fo2–Fc2)2/∑n + r – p)]½

Compound	**2·Me₂CO**	**2·MeCN**	**2·MeCOOH**	**2·MeNO₂**
Empirical formula	$C_{35.5}H_{37}Cl_2FeN_{10}O_{16.5}$	$C_{38}H_{32}Cl_2FeN_9O_{16}$	$C_{36}H_{38}Cl_2FeN_{10}O_{18}$	$C_{35}H_{37}Cl_2FeN_{11}O_{18}$
Formula weight	994.50	997.47	1025.51	1026.50
Temperature/K	120.0	119.9(3)	120.00(10)	120.00(10)
Crystal system	monoclinic	monoclinic	monoclinic	monoclinic
Space group	$P2_1/c$	$P2_1/c$	$P2_1/c$	$P2_1/c$
a/Å	17.994(4)	17.6932(6)	17.7352(4)	17.8825(15)
b/Å	13.106(3)	13.8187(4)	13.3438(3)	13.2969(9)
c/Å	19.284(4)	19.6628(6)	19.5166(5)	19.5412(17)
α/°	90	90	90	90
β/°	108.855(6)	110.651(4)	108.777(3)	108.607(9)
γ/°	90	90	90	90
Volume/Å^3	4303.9(14)	4498.6(3)	4372.87(19)	4403.7(6)
Z	4	4	4	4
ρ_{calc}g/cm^3	1.535	1.473	1.558	1.548
μ/mm^{-1}	0.559	0.533	0.555	0.552
F(000)	2048.0	2044.0	2112.0	2112.0
Crystal size/mm^3	0.19 × 0.16 × 0.12	0.6 × 0.4 × 0.1	0.85 × 0.5 × 0.15	0.6 × 0.55 × 0.1
Radiation	MoKα (λ = 0.71073)	MoKα (λ = 0.71073)	MoKα (λ = 0.71073)	MoKα (λ = 0.71073)
2Θ range for data collection/°	3.826 to 55.454	5.736 to 54.2	6.106 to 59.734	6.128 to 59.688
Index ranges	-23 ≤ h ≤ 23, -17 ≤ k ≤ 17, -24 ≤ l ≤ 25	-18 ≤ h ≤ 22, -17 ≤ k ≤ 17, -25 ≤ l ≤ 22	-24 ≤ h ≤ 24, -17 ≤ k ≤ 18, -25 ≤ l ≤ 25	-22 ≤ h ≤ 24, -18 ≤ k ≤ 17, -27 ≤ l ≤ 27
Reflections collected	161175	37614	41664	38470
Independent reflections	10071 [R_{int} = 0.4173, R_{sigma} = 0.1106]	9920 [R_{int} = 0.0603, R_{sigma} = 0.0793]	11456 [R_{int} = 0.0471, R_{sigma} = 0.0631]	11547 [R_{int} = 0.0577, R_{sigma} = 0.0767]
Data/restraints/ parameters	10071/1/599	9920/48/667	11456/32/626	11547/3/632
Goodness-of-fit on F^2	1.021	1.012	1.027	1.049
Final R indexes [I>=2σ (I)]	R_1 = 0.0751, wR_2 = 0.2127	R_1 = 0.0835, wR_2 = 0.2219	R_1 = 0.0782, wR_2 = 0.2039	R_1 = 0.0940, wR_2 = 0.2450

aR1(F) = ∑||Fo|–|Fc||/∑|Fo|; bwR2(F2) = [∑w(Fo2–Fc2)2/∑wFo4]½; cS(F2) = [∑w(Fo2–Fc2)2/∑n + r – p)]½

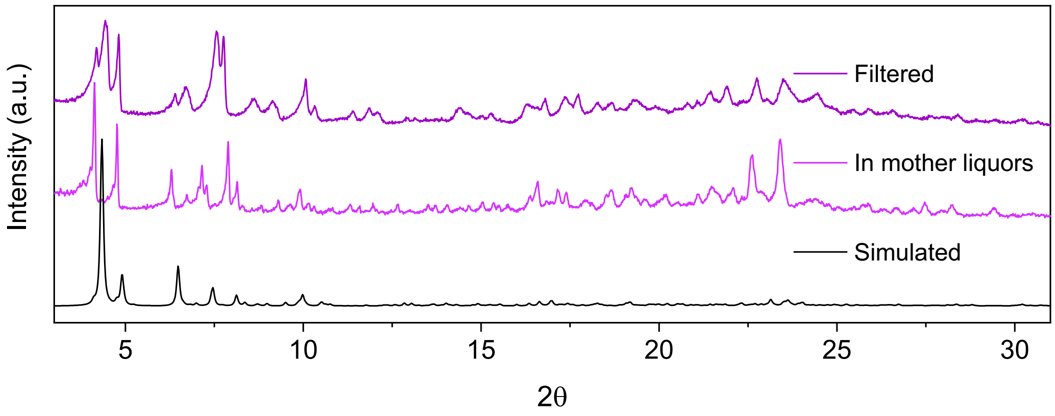

(a)

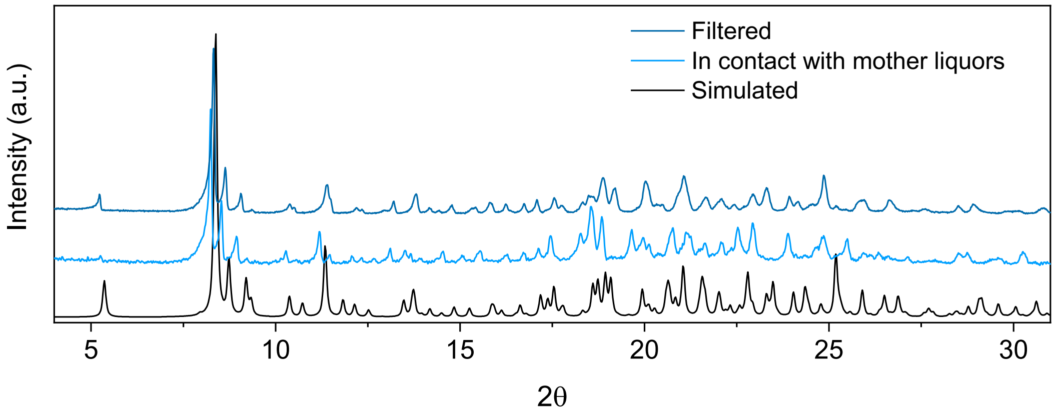

(b)

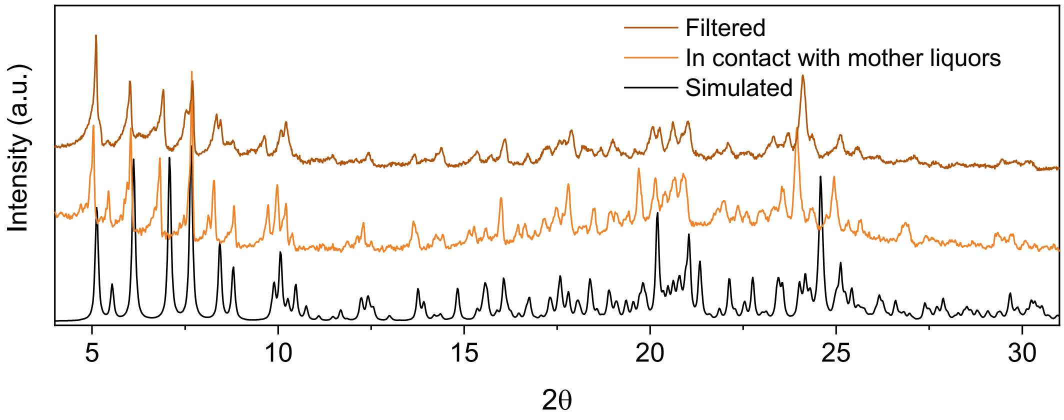

(c)

Figure S1. Experimental and simulated powder X-ray diffraction (PXRD) patterns of **1** (a), **2** (b) and **3** (c) in contact with the mother liquor and filtered.

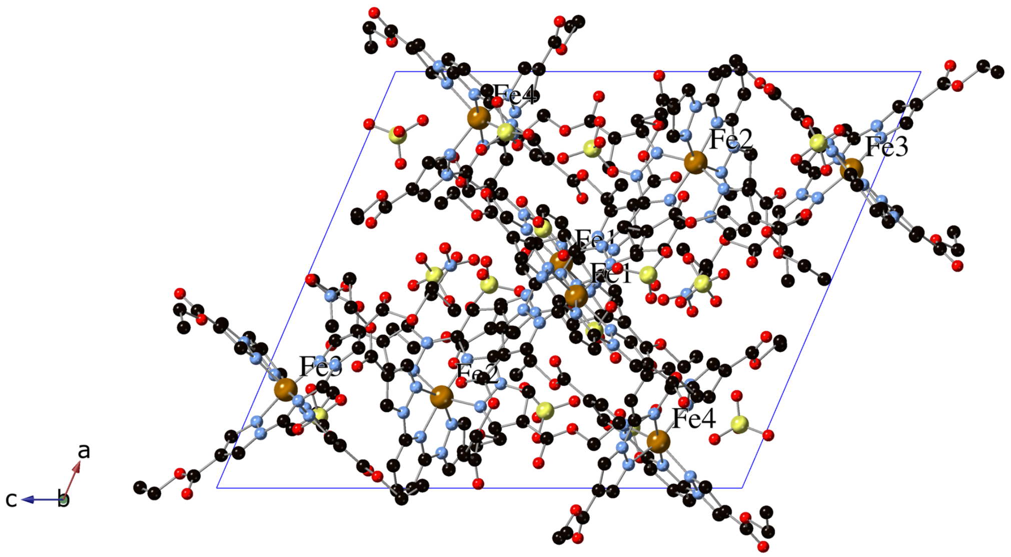

Figure S2. Projection of the structure of **1** at 120 K in the ac plane. Fe (orange), Cl (yellow), C (black), N (blue) and O (red). Hydrogen atoms have been omitted for clarity.

Intermolecular interactions in the structure of 2.

Neighboring $[Fe(1bpCOOEt_2p)_2]^{2+}$ complexes present CO···CH interactions between the free carbonyl group and the pyrazolyl and pyridine rings. This gives rise to chains of complexes along the *b* axis (**Figure S2**). These chains are linked through contacts involving O atoms from carboxylic acid groups and CH groups from pyridine and pyrazole leading to a double layer of complexes in the *bc* plane (see **Figure S3**). This network of interactions is maintained after desolvation of the compound and channels running along the *b* axis are observed.

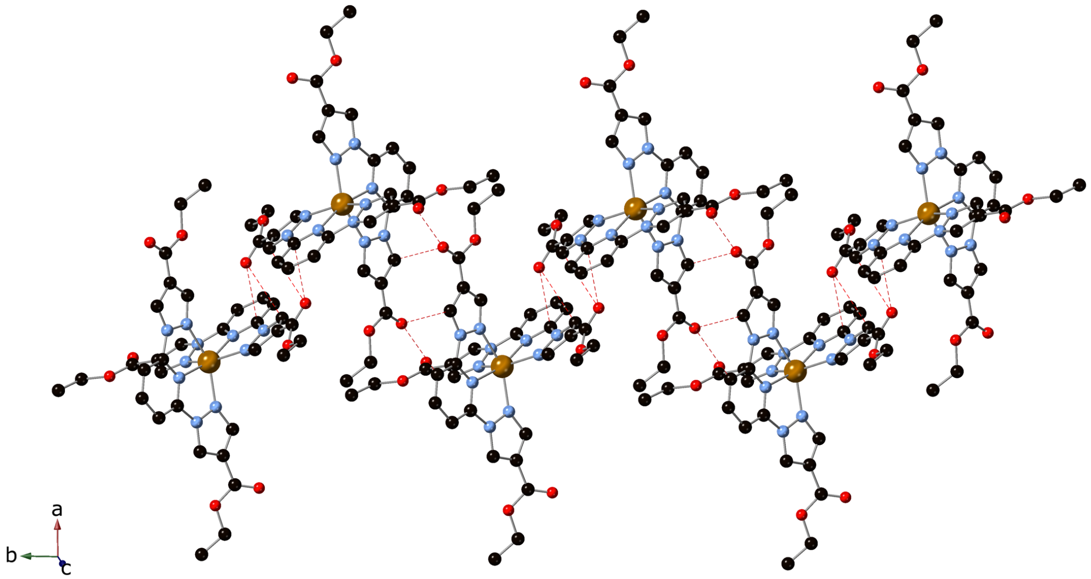

Figure S3. Intermolecular interactions between $[Fe(1bpCOOEt_2p)_2]^{2+}$ complexes of **2** at 400 K (red dashed lines) Fe (orange), Cl (yellow), C (black), N (blue) and O (red). Hydrogen atoms have been omitted for clarity.

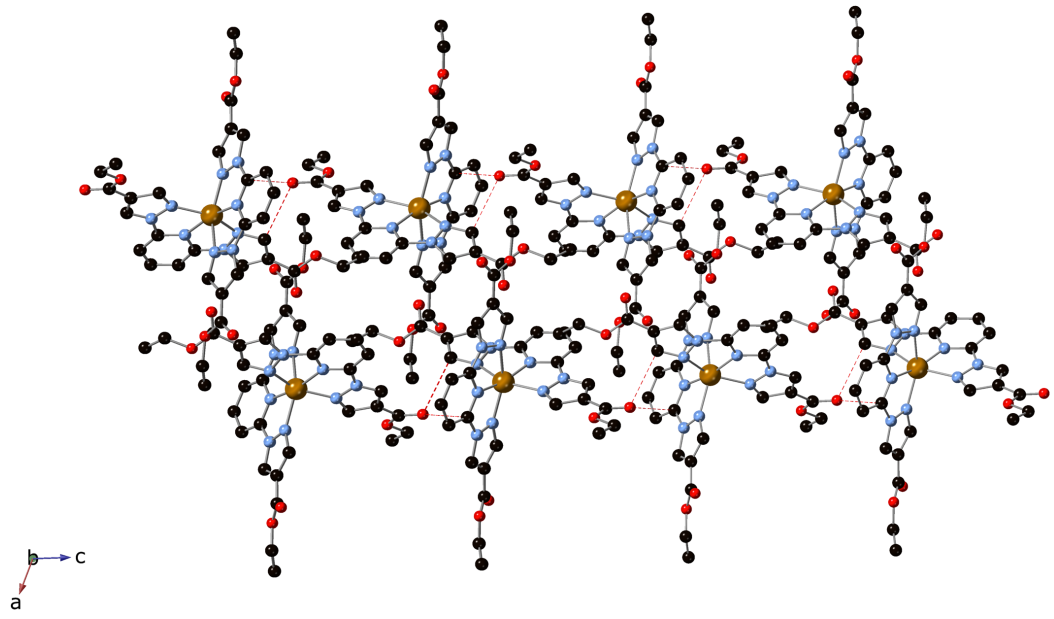

Figure S4. Intermolecular interactions between $[Fe(1bpCOOEt_2p)_2]^{2+}$ complexes of **2** at 400 K (red dashed lines) Fe (orange), Cl (yellow), C (black), N (blue) and O (red). Hydrogen atoms have been omitted for clarity.

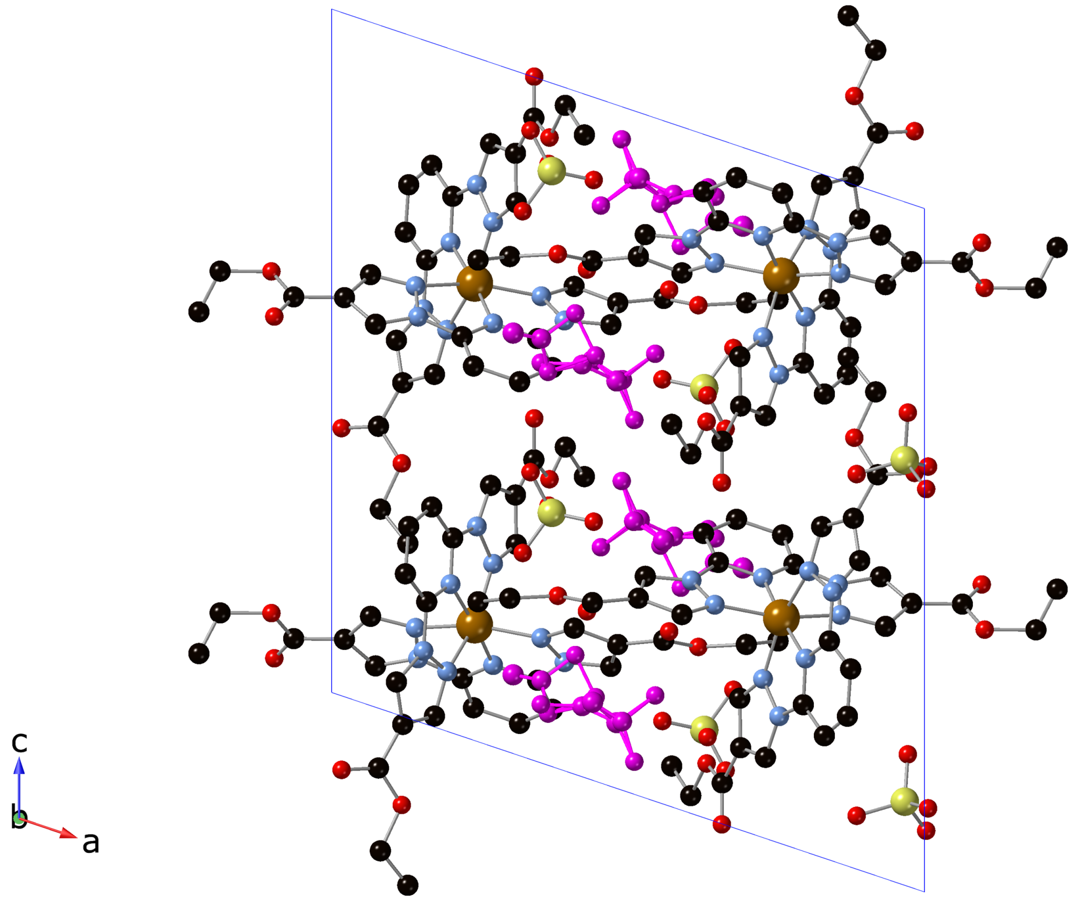

(a)

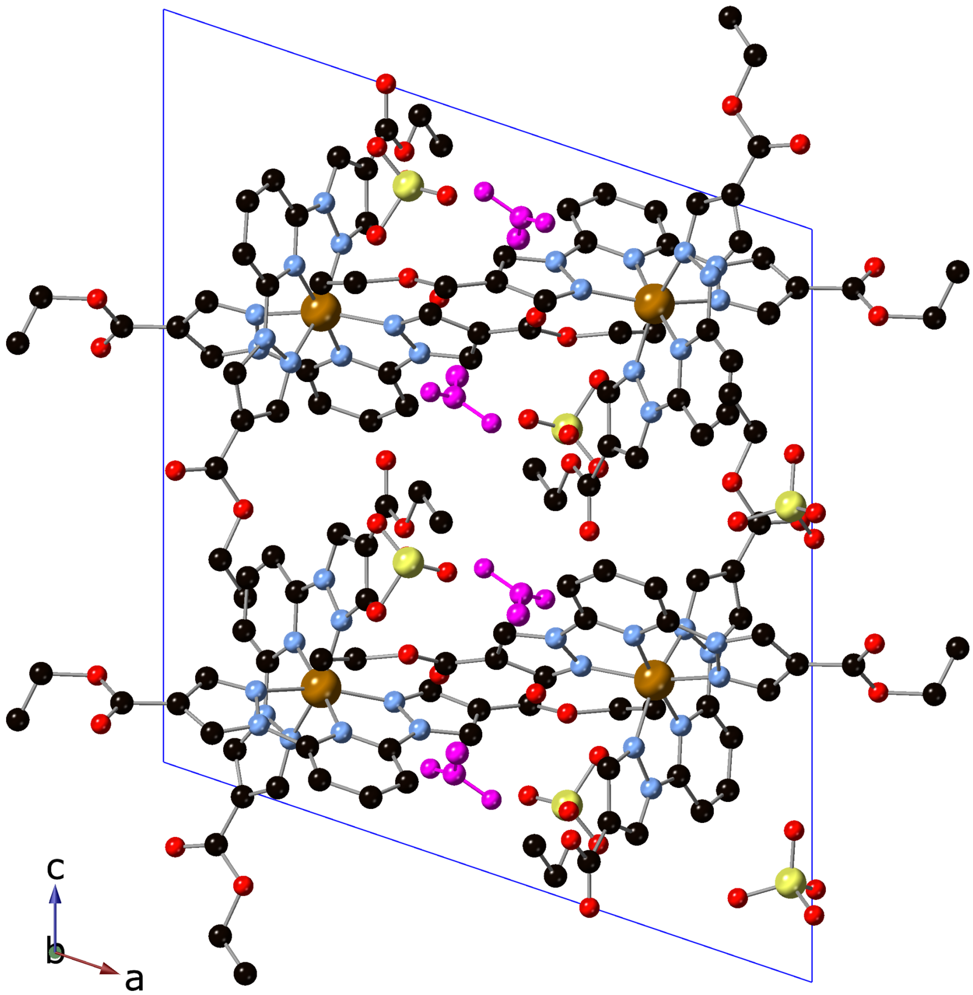

(b)

Figure S5. Projection of the structure of **2·MeNO$_2$ (a)** and **2·MeCOOH (b)** at 120 K in the *ac* plane. Fe (orange), Cl (yellow), C (black), N (blue) and O (red). MeNO$_2$ and MeCOOH solvent molecules have been colored in violet. Hydrogen atoms have been omitted for clarity.

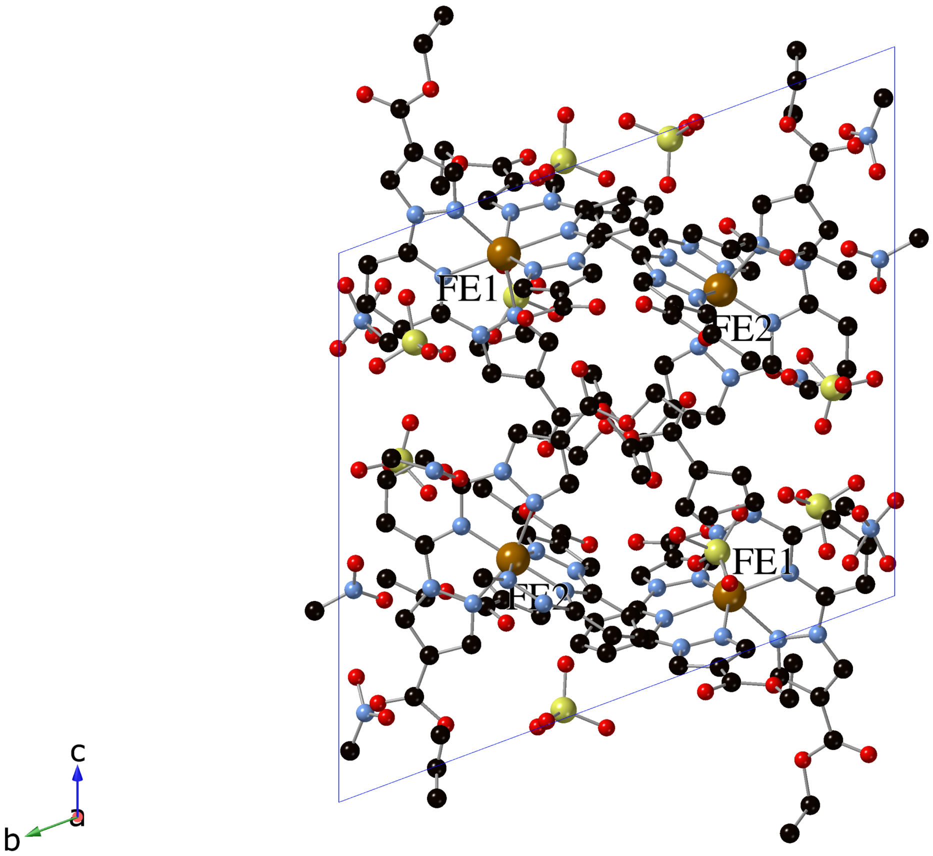

Figure S6. Projection of the structure of **3** at 120 K in the *bc* plane. Fe (orange), Cl (yellow), C (black), N (blue) and O (red). Hydrogen and disordered atoms have been omitted for clarity.

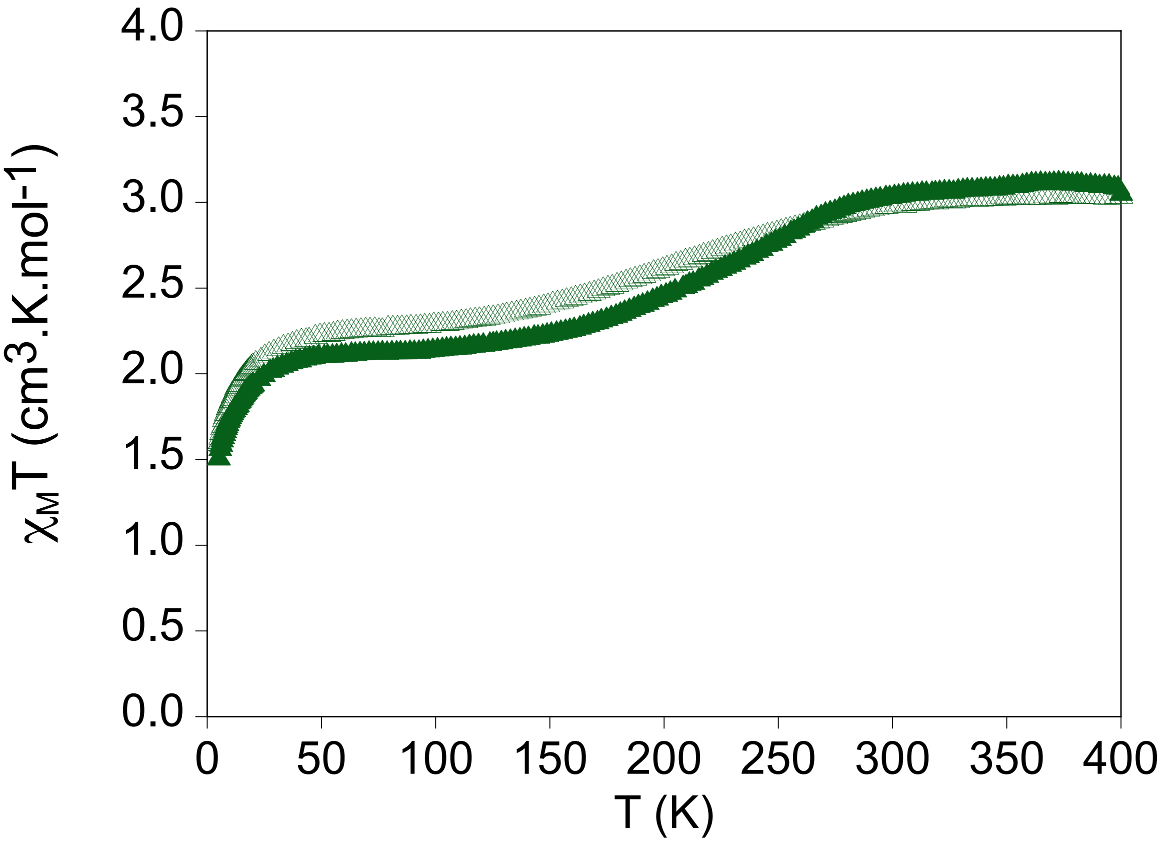

Figure S7. Thermal variation of $\chi_M T$ for **1** (green triangles). Full triangles: first heating from 5 to 400 K; empty triangles: first cooling from 400 to 5 K after first heating to 400 K.

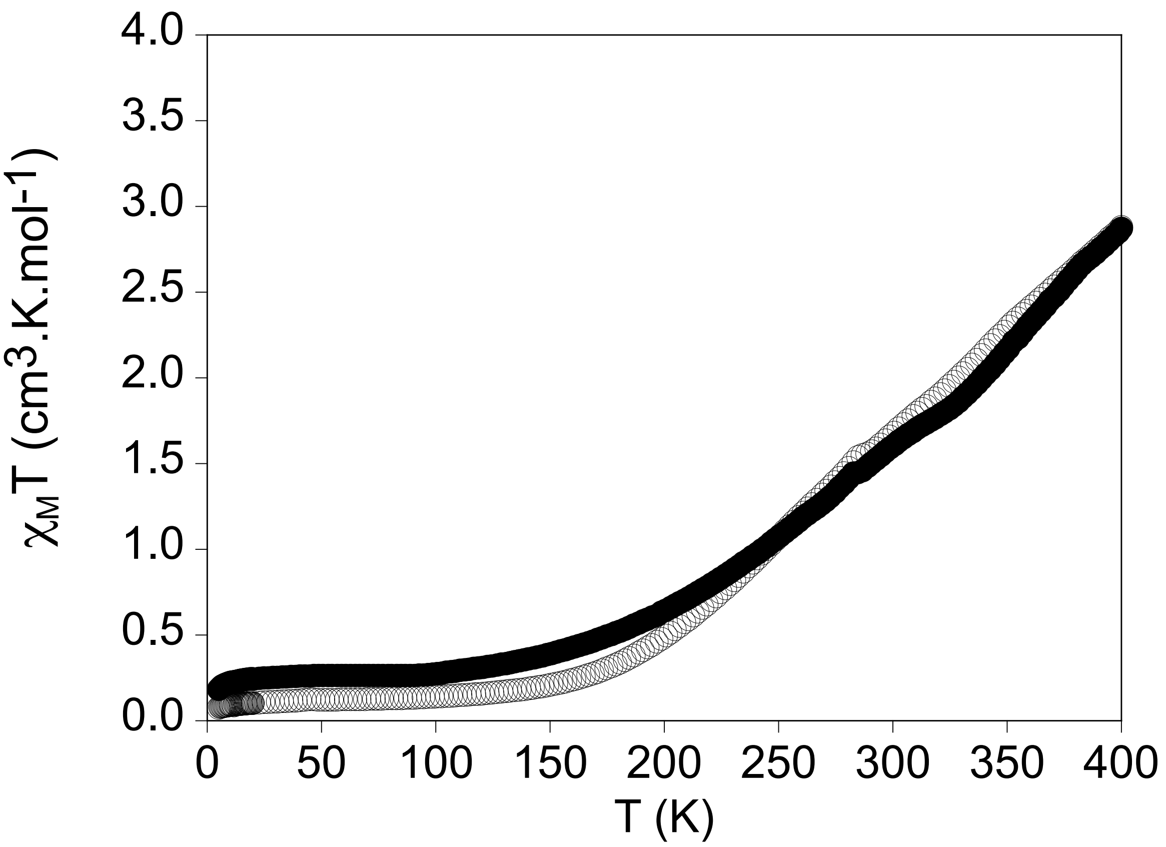

Figure S8. Thermal variation of $\chi_M T$ for **2** (black circles). Full circles: first heating from 5 to 400 K; empty circles: second heating from 5 to 400 K after first heating to 400 K.

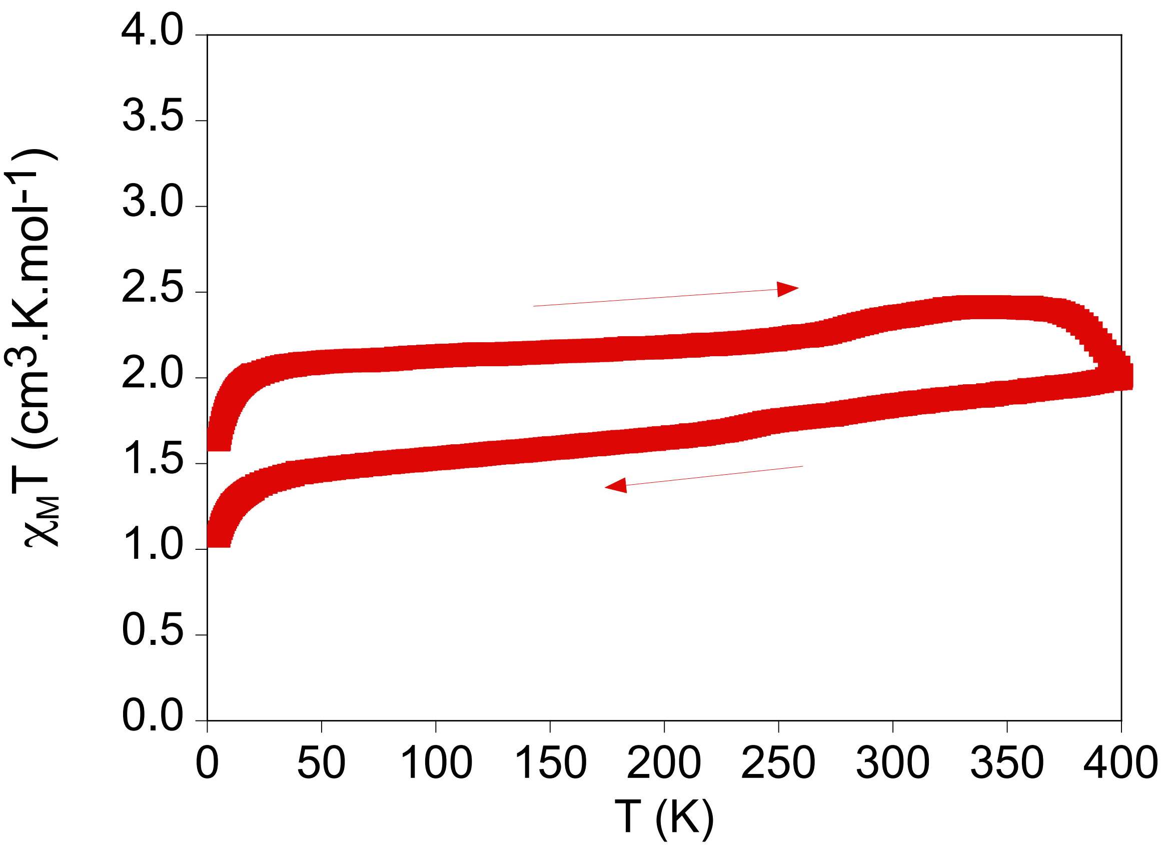

Figure S9. Thermal variation of $\chi_M T$ for a filtered sample of **3**.

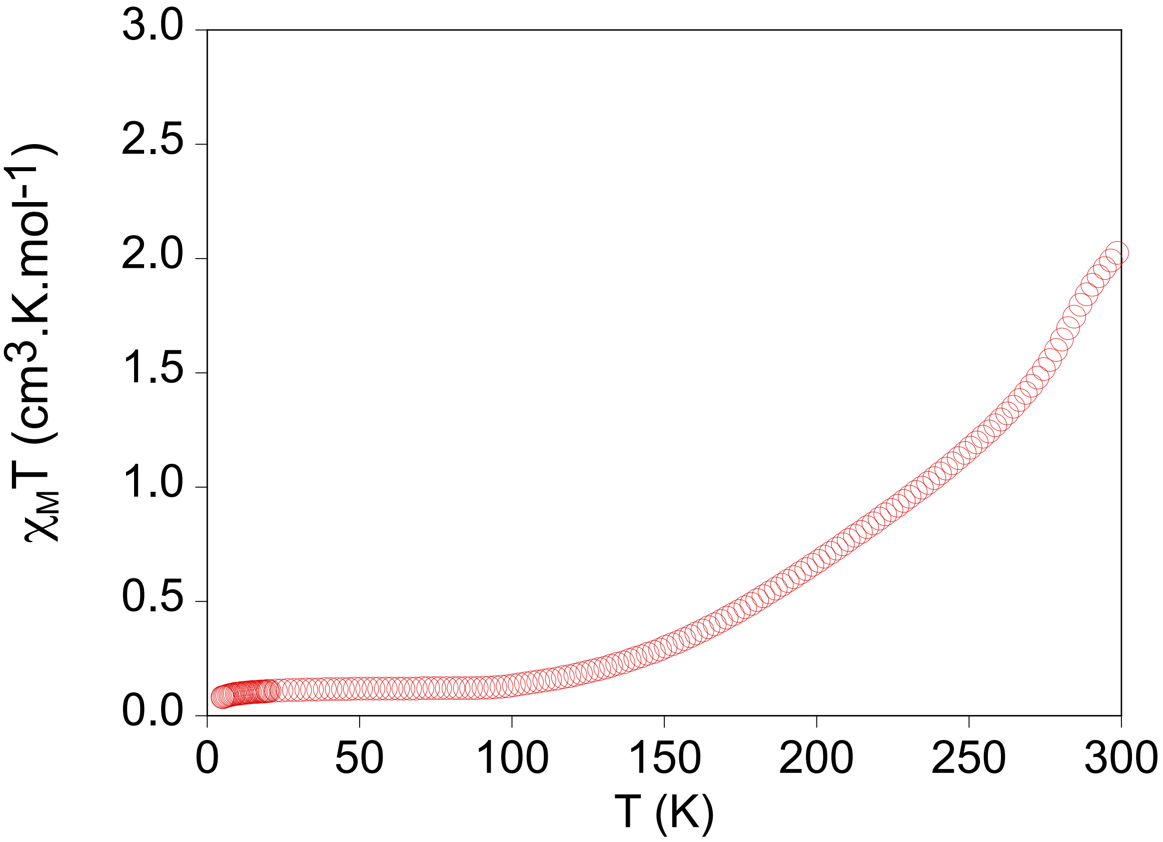

Figure S10. Thermal variation of $\chi_M T$ for **2·MeCOOH**.
